# MCT4-driven CAF-mediated metabolic reprogramming in breast cancer microenvironment is a vulnerability targetable by miR-425-5p

**DOI:** 10.1038/s41420-024-01910-x

**Published:** 2024-03-14

**Authors:** Alessandra Affinito, Cristina Quintavalle, Rosario Vincenzo Chianese, Giuseppina Roscigno, Danilo Fiore, Valeria D’Argenio, Guglielmo Thomas, Alessia Savarese, Francesco Ingenito, Lorenza Cocca, Silvia Nuzzo, Maxim V. Berezovski, Maria Patrizia Stoppelli, Gerolama Condorelli

**Affiliations:** 1 Department of Molecular Medicine and Medical Biotechnology, “Federico II” University of Naples, Naples, Italy; 2 AKA Biotech, Naples, Italy; 3 Institute Experimental Endocrinology and Oncology “Gaetano Salvatore” (IEOS), CNR, Naples, Italy; 4 Department of Human Sciences and Quality of Life Promotion, San Raffaele Open University, Roma, Italy; 5 CEINGE-Biotecnologie Avanzate Franco Salvatore, Napoli, Italy; 6 Mediterranea Cardiocenter, Naples, Italy; 7 IRCCS SYNLAB SDN, Naples, Italy; 8 Department of Chemistry and Biomolecular Sciences and John L. Holmes Mass Spectrometry Facility, University of Ottawa, Ottawa, ON Canada; 9 IGB, CNR, Naples, Italy

**Keywords:** Cancer microenvironment, Breast cancer, Targeted therapies

## Abstract

Multiple oncogenic alterations contribute to breast cancer development. Metabolic reprogramming, deeply contributing to tumor microenvironment (TME) education, is now widely recognized as a hallmark of cancer. The reverse Warburg effect induces cancer-associated fibroblasts (CAFs) to produce and secrete L-lactate, enhancing malignant characteristics such as neoangiogenesis, metastatic dissemination, and treatment resistance. Monocarboxylate transporter (MCT) 4 is involved in lactate efflux from CAFs into stromal and epithelial cells. Here, we first assess the expression of miR-425-5p and its target MCT4 in breast cancer CAFs and normal fibroblasts. We analyzed the metabolic changes induced by miR-425-5p in CAFs and its role in the education of breast cancer epithelial cells. We show that miR-425-5p-induced MCT4 knockdown decreased lactate extrusion from CAFs and its availability in the TME. miR-425-5p overexpression induced profound metabolic transformation in CAFs, ultimately influencing breast cancer metabolism. Furthermore, miR-425-5p impaired the capacity of CAFs to sustain vessel formation and breast cancer cell migration, viability, and proliferation. These findings emphasize the key role of miR-425-5p in breast cancer metabolism and aggressiveness, and its possible importance for breast cancer therapy and monitoring.

## Introduction

Cancer-associated fibroblasts (CAFs) are critical players in breast cancer (BC) development, migration, and proliferation [1–3]. Thanks to an extensive desmoplastic reaction that activates fibroblasts, CAFs account for up to 80% of the tumor mass in BC [4] and participate in tumor development and metastasis by secreting growth factors and chemokines [4]. Besides the canonical mitochondrial oxidative phosphorylation (OXPHOS), most cancer cells can generate energy by converting glucose to lactate [5]. By supplying key metabolic intermediates for adenosine triphosphate (ATP) generation, CAFs are of paramount importance for BC cell metabolic rewiring. In parallel, CAFs undergo metabolic reprogramming themselves, becoming more glycolytic, whereas cancer cells rely more on their mitochondrial energy generation pathways via OXPHOS. These findings are at odds with traditional Warburg theory, claiming that tumor cells are highly glycolytic and have decreased mitochondrial function, and have propelled a “reverse Warburg hypothesis” [6, 7], sustaining that anabolic cancer cells and catabolic CAFs are biologically connected via monocarboxylate transporters (MCTs) and that CAFs generate huge quantities of energy-rich fuels, harnessing aerobic glycolysis to ultimately feed mitochondrial OXPHOS in neighboring anabolic cancer cells [8].

MCTs are proton-linked membrane transporters that vehiculate single-carboxylate molecules—like lactate, pyruvate d-β-hydroxybutyrate, and acetoacetate—in and out of cells [9]. MCT1 and MCT4 have been implicated in the metabolic interaction between cancer cells and fibroblasts [10–12] and are upregulated in multiple carcinomas, (i.e. breast, lung, stomach, and colon tumors) [13]. MCT1 is upregulated in OXPHOS cancer cells with enhanced lactate absorption, while MCT4 expression is increased in lactate-exporting glycolytic cells and is linked to hypoxia control [14]. Recently, improved understanding of the heterogeneity of tumor metabolism has propelled the identification of novel biomarkers and targetable vulnerabilities to be translated in (pre)clinical settings [15]. Indeed, metabolic dependencies may be used as predictors of cancer prognosis and to develop patient-tailored therapeutics [16–18]. For instance, higher MCT1 expression in carcinoma cells has been linked to lower progression-free survival and increased probability of recurrence in triple-negative BC [19]. MCT1/4 have been associated with metabolic transitions and cancer aggressiveness and chemo refractoriness [20, 21]. Also, MCT4 over-expression increases tumor growth and progression [22–24], and provides a tumor-promoting microenvironment [25]. As a consequence, MCTs inhibitors have been proven to induce cytotoxicity [26, 27], enhance immune response [28, 29] and modulate BC symptoms [30] so much so that they are currently in pre-clinical trials on BC [31]. MCT4 expression in BC is associated with lower survival [32] and higher ulcerated tumors [33], and its expression can be considered a predictor of invasiveness [34]. The tight interweaving of signaling molecules, oncogenes, and tumor suppressor genes involved in cancer cell metabolic reprogramming is also controlled by microRNAs (miRNAs) [35]. Recently, it has been shown that miR-425-5p downregulates MCT4 and leads to the accumulation of lactate in human umbilical vein endothelial cells (HUVECs) [36].

In this study, we demonstrate that BC CAFs rely on MCT4 to maintain lactate extrusion into the tumor microenvironment (TME), promoting metabolic-related transformation of tumor cells. Also, we show that miR-425-5p overexpression downregulates the level of MCT4 in BC CAFs, ultimately hindering lactate effusion. Accordingly, conditioned medium from CAFs overexpressing miR-425-5p induced apoptosis and impaired migration in MDA-231-MB BC cells. miR-425-5p also hampered ATP production and glycolysis in CAFs. Ultimately, we prove evidence that MCT4 is a metabolic vulnerability for BC–CAF crosstalk and that miR-425-5p, by downregulating MCT4, modulates the protumorigenic contribution of CAFs to BC environment and metabolism.

## Results

### MCT4 expression level and L-lactate secretion are higher in CAFs than in normal fibroblasts (NFs)

**Fig. 1:**
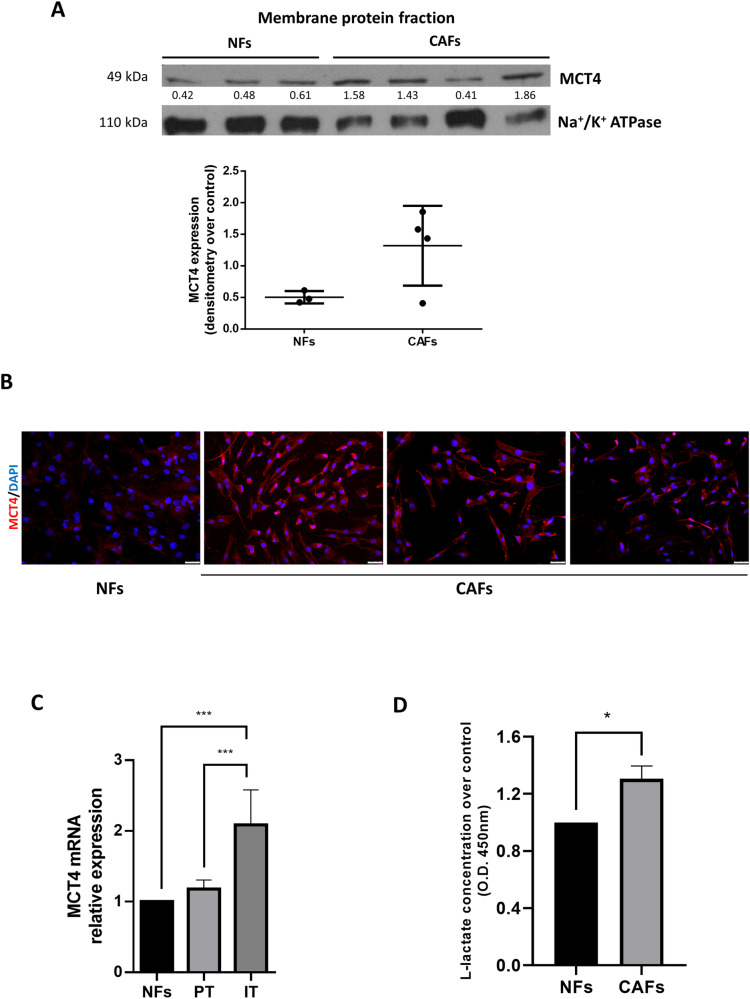
MCT4 is highly expressed in Cancer-Associated Fibroblasts. Representative Western blot analysis of MCT4 performed on plasmamembrane fractions of Cancer-Associated Fibroblasts (CAFs) (*n* = 4) and Normal Fibroblasts (NFs) (*n* = 3). Na + /K+ ATPase was used for data normalization of the fractionation in the sample preparation. Densitometry data are expressed after normalization on Na + /K+ ATPase band intensity **A**. A representative confocal microscopy image of MCT4 expression in CAFs (*n* = 3) (DAPI blue, MCT4 red) **B**. Three different subpopulations of two primary fibroblasts were collected at different growing distances from the tumor (intratumoral, IT; peritumoral, PT; normal, NF) and MCT4 gene expression was evaluated by quantitative real-time PCR. Values are expressed as a mean of two biological replicates of technical duplicates **C**. Culture media from NFs (*n* = 2) and CAFs (*n* = 2) were collected and assessed by a colorimetric L-lactate assay. Values are expressed as mean of two technical replicates **D**. Graph shows mean ± SD. * *P values* < 0.05; ***p < 0.001. Bar = 100 μm.

Previous studies showed that the MCT4 was upregulated in CAFs in several tumors [11, 37]. To address the lactate transporter role in BC TME, we first evaluated MCT4 expression through Western blotting, immunofluorescence, and real-time PCR in freshly isolated BC CAFs and NFs. CAFs from BC patients had increased expression of MCT4 on the cell membrane when compared to NFs (Fig. 1A, B). To check if MCT4 expression correlated with fibroblasts activation, we assessed MCT4 mRNA level in three subpopulations of fibroblasts collected from the same patient at increasing distance from the tumor: i) intratumoral, ii) peritumoral, and iii) normal fibroblasts [38]. MCT4 expression was inversely correlated with the distance from the tumor and was higher in fibroblasts within the tumoral mass than in NFs (Fig. 1C). Given the involvement of MCT4 in lactate secretion, we performed a metabolic assay to measure L-lactate in media harvested from cultured CAFs and NFs. As expected, CAFs secreted a higher amount of lactate (Fig. 1D).

Thus, CAFs had increased expression of MCT4, a transporter involved in lactate extrusion into the TME, ultimately maintaining the reverse Warburg effect to fuel tumor growth and metastasis [39].

### MCT4 is a direct target of miR-425-5p

**Fig. 2:**
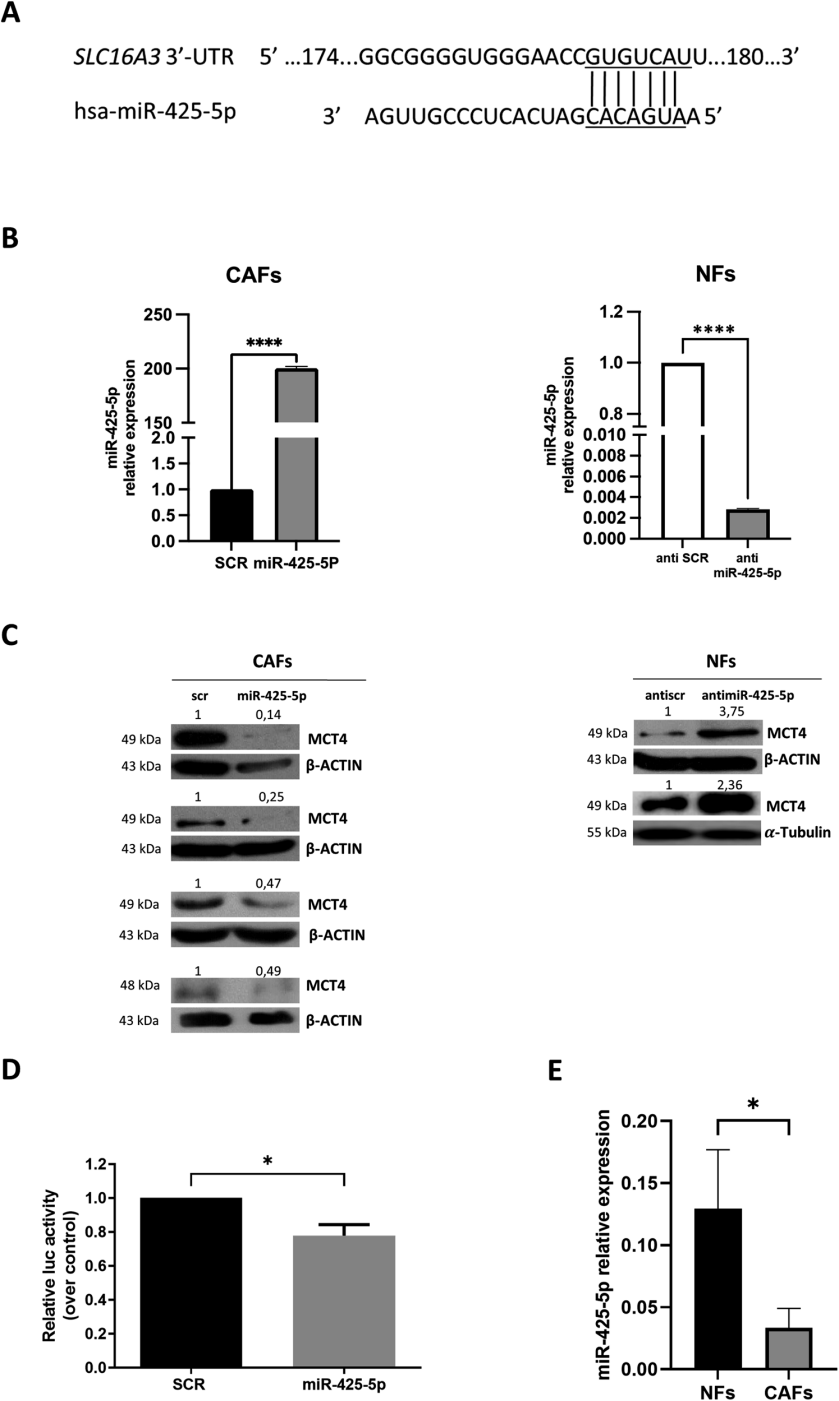
MCT4 is a direct target of miR-425-5p. Bioinformatics predictions (TargetScan and miRANDA) indicated miR-425-5p as a putative miRNA targeting MCT4 mRNA **A**. Representative Real Time PCR for miR-425-5p upon miR-425-5p and anti miR-425-5p transfection in Cancer-Associated Fibroblasts (CAF *n* = 1) and Normal Fibroblasts (NF *n* = 1) respectively. Data are expressed as mean of two technical replicates **B**. Representative Western Blot analysis of MCT4 upon miR-425-5p and anti-miR-425 transfection in CAFs (*n* = 4) and NFs (*n* = 2) respectively **C**. Dual luciferase assay of the predicted binding site for miR-425-5p on the 3’UTR region of SLC16A3. Data are expressed as mean of two technical replicates **D**. Quantitative Real Time PCR for miR-425-5p performed on CAFs (*n* = 3) and NFs (*n* = 2), to evaluate miR-425-5p levels. Values are expressed as mean of two technical replicates **E**. Graph shows mean ± SD over control. * *P values* < 0.05.

Given the role of MCT4 in subverting tumor metabolism and maintaining a hostile TME, we investigated possible miRNAs interfering with the expression of the MCT4 gene (SLC16A3). Bioinformatics algorithms (TargetScan and miRANDA) predicted a putative binding site for hsa-miR-425-5p on the 3’-UTR of SLC16A3 (Fig. 2A). To validate whether miR-425-5p could affect MCT4 expression, we transiently transfected miR-425-5p in CAFs (Fig. 2B, left panel), observing a consistent reduction of MCT4 protein level (Fig. 2C, left panel). Coherently, inhibition of miR-425-5p by anti-miR-425-5p in NFs (Fig. 2B, right panel) resulted in enhanced MCT4 expression (Fig. 2C, right panel). Furthermore, to validate MCT4 as a direct target of miR-425-5p, a dual luciferase assay was performed. Direct interaction of miR-425-5p on SLC16A3 3’-UTR mRNA was demonstrated by a reduction in luciferase activity (Fig. 2D). Of note, endogenous expression of miR-425-5p was lower in CAFs than in NFs (Fig. 2E).

### miR-425-5p modulates metabolism in CAFs

**Fig. 3:**
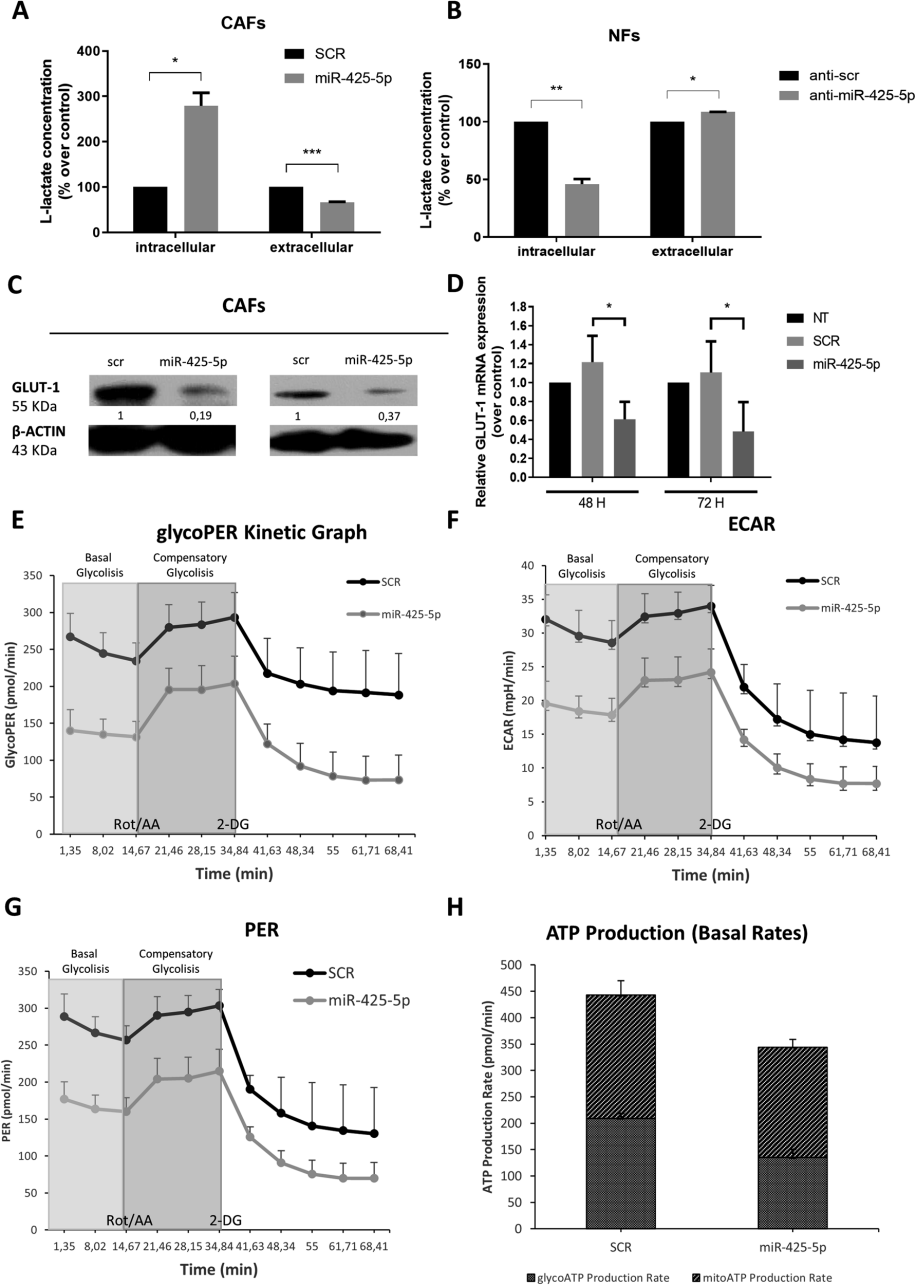
miR425-5p-induced metabolic changes in Cancer-Associated Fibroblasts. L-lactate assessed with a colorimetric assay in Cancer-Associated Fibroblasts (CAFs *n* = 2) and culture media 72 h after miR-425-5p transfection. Values are expressed as a mean of two biological replicates of technical duplicates **A**. L-lactate assessed in Normal Fibroblasts (NFs *n* = 2) and culture media 48 h after antimiR-425-5p transfection. Values are expressed as a mean of two biological replicates of technical duplicates **B**. Representative Western blot **C** and real-time analysis **D** of GLUT-1 expression in CAFs (*n* = 2) upon miR-425-5p transfection. Data are expressed as mean of two technical replicates. Metabolic changes in CAFs transfected with miR-425-5p, as assessed by glycolytic proton efflux rate (glycoPER) kinetics **E**, extracellular acidification rate (ECAR) **F**, proton efflux rate (PER) **G**, and ATP production **H**. Data are presented as a mean of two biological replicates of eight technical replicates. Data are mean percentage ± SD over control. * *P values* < 0.05; ** *P values* < 0.01.

Having demonstrated that MCT4 is directly downregulated by miR-425-5p, we wondered whether transient overexpression of miR-425-5p affected lactate secretion. Therefore, we performed a metabolic L-lactate assay to determine lactate concentration in culture media, as well as the level of intracellular lactate, in CAFs overexpressing miR-425-5p. Upon miRNA transfection, CAFs reduced their secretion of lactate accumulating the metabolite intracellularly (Fig. 3A). Coherently, NFs had enhanced lactate secretion and reduced intracellular lactate upon transfection with anti-miR-425-5p (Fig. 3B).

In parallel, we explored miR-425-5p’s effect on GLUT1 (SLC2A1), a glycolytic marker and crucial transporter for the intake of metabolic biosynthetic building components in CAFs [5]. GLUT1 controls glycolytic rate, glucose uptake, and lactate secretion in BC [40] and is involved in the metabolic shift from OXPHOS to aerobic glycolysis [41]. GLUT1 and MCT4 are often co-regulated [12, 42], indeed both are upregulated in invasive breast cancer (BRCA) compared to normal cells (Supplementary Fig. 1A, B) and correlate with each other (Supplementary Fig. 1C). Of note, we found that miR-425-5p also impaired GLUT1 mRNA and protein (Fig. 3C, D).

The mechanism by which GLUT1 is downregulated has not been investigated in this paper, but according to TargetScan the GLUT1 gene seems not to be directly inhibited by miR-425-5p (data not shown). To further unravel the impact of miR-425-5p in reprogramming CAF metabolism, we performed a Seahorse XF Glycolytic Rate Assay for real-time measurements of functional changes of glycolysis in CAFs transfected with miR-425-5p. We found that the glycolytic proton efflux rate (glycoPER), a direct measurement of extracellular lactate accumulation [43], was significantly reduced upon miR-425-5p transfection during basal and compensatory glycolysis (Fig. 3E and Supplementary Fig. 2A, B). Additionally, extracellular acidification rate (ECAR) and PER decreased in miR-425-5p-transfected CAFs as a measure of reduced extracellular acidification resulting from the decreased proton extrusion by the MCT4/H^+^ symporter (Fig. 3F, G). Moreover, miR-425-5p mediated alteration of the bioenergetic profile of CAFs and involved a decreased rate of ATP production in the glycolytic pathway (glycoATP) (Fig. 3H). Collectively, these findings demonstrate a reduced lactate extrusion, extracellular acidification, and decreased ATP production, in CAFs overexpressing miR-425-5p.

**Fig. 4:**
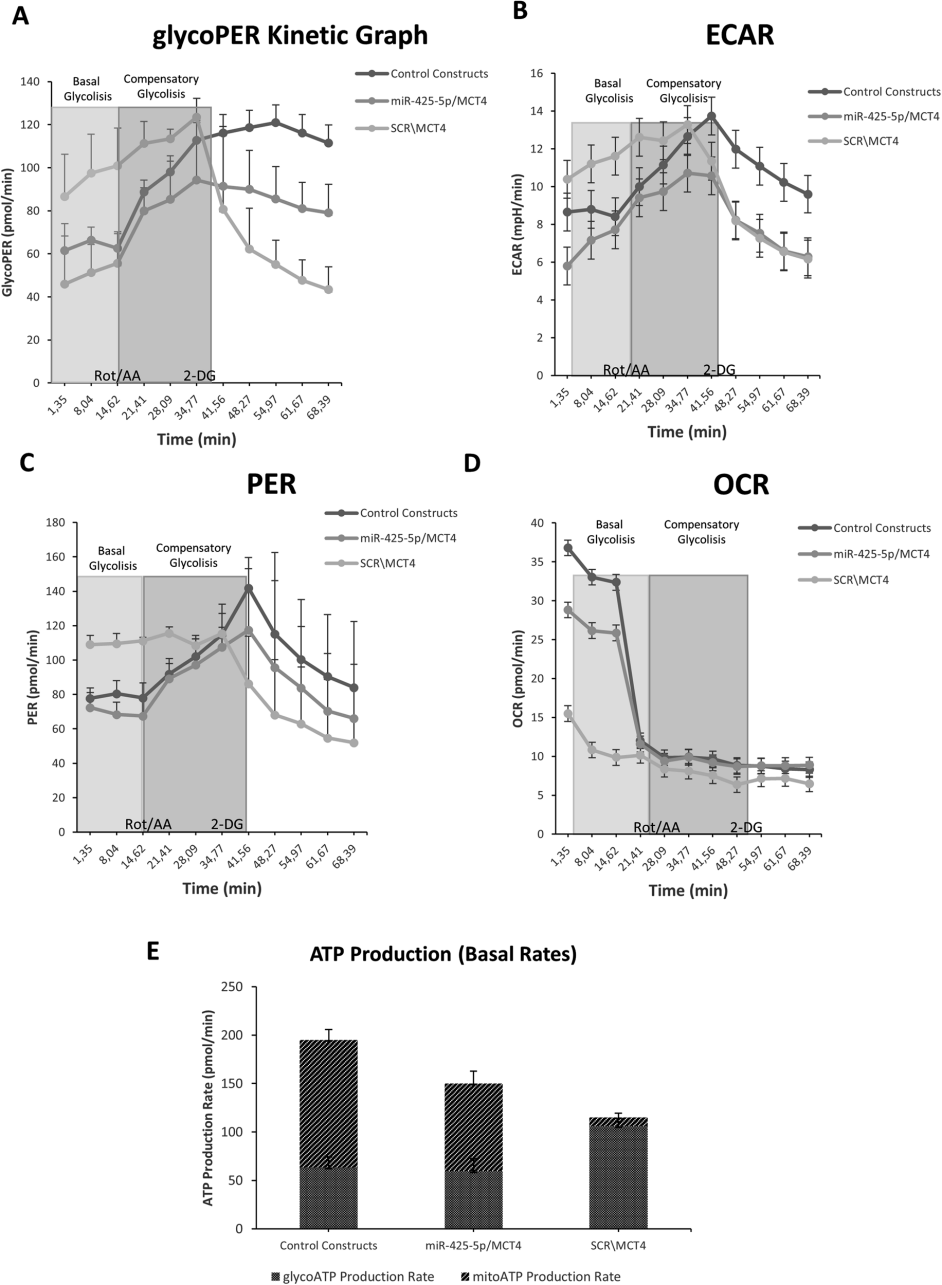
miR-425-5p reverts MCT4-mediated metabolic shift resulting in reprogramming of Cancer-Associated Fibroblasts. Metabolic changes in MCT4-transfected MS-5 cells with or without miR-425-5p overexpression, as assessed by glycolytic proton efflux rate (glycoPER) kinetics **A**, proton efflux rate (PER) **B**, extracellular acidification rate (ECAR) **C**, oxygen consumption rate OCR **D**, and ATP production **E**. Data are presented as a mean of biological duplicates of eight technical replicates ± SD over control.

Because plasmids are very difficult to transfect into primary CAFs [44–47], we exogenously expressed MCT4 in a continuous normal bone-marrow mesenchymal fibroblastoid cell line (MS-5). We first assessed the ability of miR-425-5p to reduce glycoPER, ECAR, and PER also in MS-5 cells (Supplementary Fig. 2C–E). Then, to perform a rescue assay, we simultaneously overexpressed miR-425-5p together with MCT4 DNA (deprived of 3′UTR). We found that exogenous MCT4 overexpression in MS-5 cells increased glycoPER at basal and compensatory levels (Fig. 4A and Supplementary Fig. 2F, G). Remarkably, co-transfection with miR-425-5p reduced the accumulation of extracellular lactate. Also, proton efflux and extracellular acidification rate were increased by MCT4 and rescued by miR-425-5p co-transfection (Fig. 4B, C). In addition, oxygen consumption rate (OCR), a key parameter of mitochondrial function, was strongly decreased after MCT4 overexpression, while glycoATP rate increased, ultimately proving the metabolic shift from OXPHOS to glycolysis (Fig. 4D, E). This phenotype was reverted in cells simultaneously transfected with miR-425-5p.

These findings demonstrated the presence of a hitherto unknown miR-425-5p–MCT4 axis involved in lactate extrusion from CAFs, and its involvement in reprogramming BC metabolism and environment.

### miR-425-5p overexpression in CAFs reduces BC cell aggressiveness

To mechanistically dissect the effects of miR-425-5p-mediated reprogramming of CAF metabolism in BC, we assessed whether conditioned medium (CM) collected from miR-425-5p-overexpressing CAFs could threaten the lactate-mediated interplay between BC cells and the TME. To this aim, we collected CM from CAFs overexpressing miR-425-5p, in order to detect any variations in lactate concentration due to MCT4 knockdown. Thereafter, we used the CM to culture MDA-MB-231 cells—a triple-negative continuous BC cell line—to evaluate how lactate affects the protumorigenic phenotype of BC cells.

**Fig. 5:**
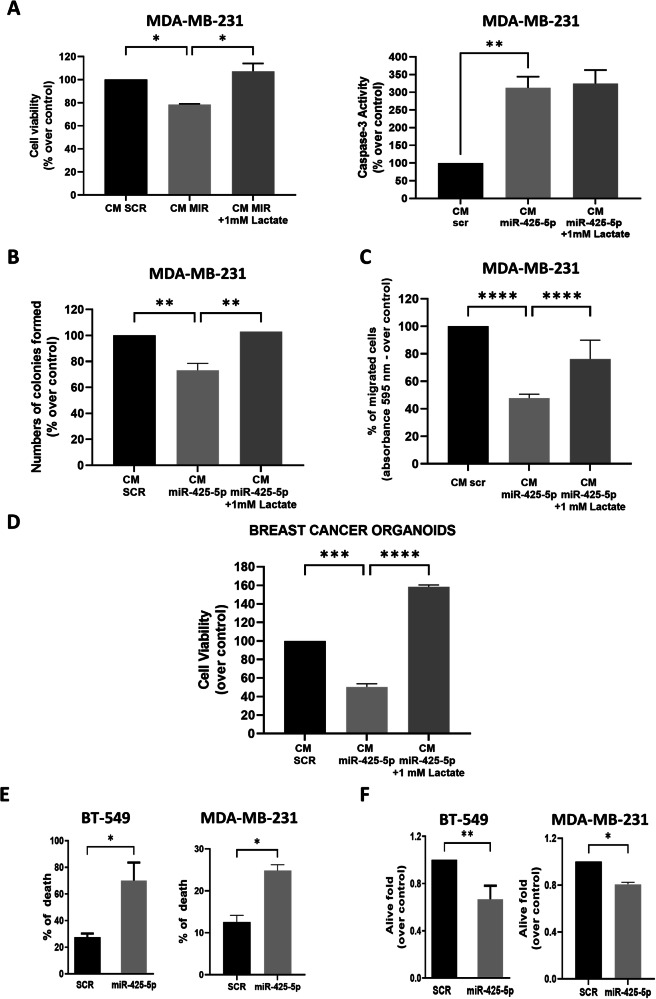
Metabolic reprogramming of breast cancer cells by conditioned medium (CM) from miR-425-5p-overexpressing Cancer-Associated Fibroblasts. Cell viability and caspase 3-activation **A**, colony formation assay **B**, and migration assay **C** of MDA-MB-231 cells treated with CM from Cancer-Associated Fibroblasts (CAFs) overexpressing miR-425-5p or a scrambled sequence. 1 mM of lactate was used in rescue experiments. Cell viability in luminal B-type 3D BC organoids upon treatment with CM from CAFs overexpressing miR-425-5p or a scrambled sequence. Data are expressed as mean of two biological replicates **D**. Cell death **E** and cell viability **F** of CMDFA-labelled BT-549 or MDA-MB-231 is shown upon co-culture with CAFs overexpressing miR-425-5p/scrambled sequence. Data are Values are expressed as biological means (*n* = 2) of technical triplicates. * *P values* < 0.05; ** *P values* < 0.01; *** *P values* < 0.001, **** *P values* < 0.0001.

CM from CAFs overexpressing miR-425-5p reduced the metabolic activity of MDA-MB-231 cells, concomitantly upregulating apoptosis (Fig. 5A), decreasing colony formation (Fig. 5B), and impeding transwell migration (Fig. 5C). Similar results were obtained using an alternative triple negative BC cell line, BT-549 (Supplementary Fig. 3A).

To translate our findings to a more complex and reliable model representative of BC heterogeneity and 3D architecture, we developed patient-derived BC organoids. Of note, BC organoids exposed to CM from CAFs overexpressing miR-425-5p had decreased viability (Fig. 5D). Furthermore, the addition of exogenous lactate to the CM at least partially reverted the effects shown in Fig. 5A–D and Supplementary Fig. 3A, demonstrating a direct link between lactate and the observed phenotypes.

In addition, BC cells co-cultured with miR-425-5p-overexpressing CAFs were characterized by increased cell death (Fig. 5E), decreased cell proliferation (Fig. 5F), and arrest in the G1 phase of the cell cycle (Supplementary Fig. 2E).

To corroborate these findings, MDA-MB-231 and BT-549 cells were exposed to CM from NFs transfected with anti miR-425-5p. As expected BC cells had increased viability and reduced caspases 3/7 activation **(**Supplementary Fig. 3B, C).

Together, the above demonstrates a role of miR-425-5p in TME and in, ultimately reducing BC cell aggressiveness.

### Conditioned medium from miR-425-5p-overexpressing CAFs reduces tumor neoangiogenesis

**Fig. 6:**
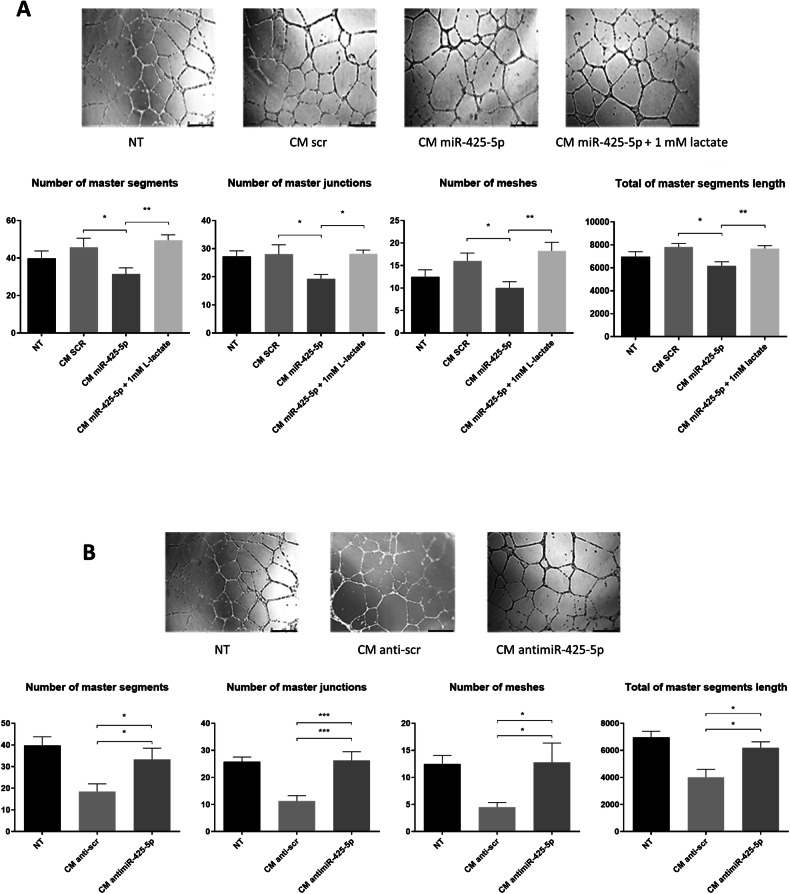
miR-425-5p overexpression in stromal fibroblasts modulates angiogenesis in the tumor microenvironment. Representative images and quantifications of tube formation by HUVECs after culture in conditioned medium (CM) from miR-425-5p-overexpressing Cancer-Associated Fibroblasts (CAFs), compared to the control. 1 mM of lactate was used in rescue experiments **A**. Representative images and quantifications of tube formation by HUVECs cultured in CM from Normal Fibroblasts (NFs) with downregulated miR-425-5p, compared to the control. 1 mM of lactate was used in rescue experiments **B**. Data are presented as mean of six different images for each experimental point. * *P values* < 0.05; ** *P values* < 0.01; *** *P values* < 0.001. Bar = 500 μm.

Given the direct role of lactate in promoting angiogenesis within the TME [48, 49], we also conducted a tube-formation assay on continuous HUVECs exposed to CAF CM. Lactate-depleted CM collected from miR-425-5p-transfected CAFs reduced the ability of HUVECs to form vessel-like structures; exogenous restoration of the lactate level reverted the effect (Fig. 6A). To corroborate these findings, HUVECs were cultured with CM collected from NFs upon anti-miR-425-5p transfection; in this setting, HUVECs were positively stimulated to form tubes (Fig. 6B).

## Discussion

CAFs from the majority of BC stroma, play a crucial role in the tumor microenvironment by supporting epithelial cells and promoting tumor progression and malignancy [50, 51].

As Warburg originally reported in 1956, cancer was characterized by a strongly increased glycolysis rate and high release of lactate into the tumor’s surroundings [52, 53]. However,- metabolic heterogeneity exist. Moreover, metabolic support of cancer-associated stroma to cancer cells have been widely documented [39]. So, the original observation of Otto Warburg appears today to be only a part of a more complex phenomenon that involves not only tumor cells but also stoma cells, like CAFs and endothelial cells, affecting cancer in many aspects, such as cell viability, invasive and migratory capability, drug resistance, angiogenesis, and cell proliferation.

In recent years, knowledge gained on tumor metabolism and metabolic heterogeneity has promoted the study of potential clinical markers and novel therapeutic targets. Indeed, metabolic markers –among which is MCT4 – can be used as diagnostic or prognostic markers for cancer [54–56]. Indeed, the interest in MCT inhibition has increased lately, with a phase I trial on AZD3965 [31], a novel MCT1 inhibitor for advanced solid cancer treatment (NCT01791595).

Moreover, the exploitation of RNA-based strategies as vaccines for the COVID-19 pandemic has strongly increased the scientific community’s interest in RNA therapies [57].

Here, and fully in line with the latest findings, we identify an RNA-based method for impairing MCT4 expression in CAFs, paving the way for innovative therapeutic strategies for breast cancer. We addressed the metabolic changes occurring in CAFs after the disruption of MCT4 expression mediated by miR-425-5p overexpression. Indeed, we found that restoring the miR-425-5p levels in CAFs reprograms their metabolism and leads to inhibition of their tumor-supporting and -promoting roles, impairing viability, proliferation, and migration of BC cells, so overall regulating BC aggressivity.

Recent approval by the FDA allows the use of anti-angiogenic agents to treat cancer [58, 59]. However, their use often leads to the enhancement of tumor invasiveness through the activation of mechanisms of resistance [60] that appear to be powered by stromal cells [61]. In this regard, we show that miR-425-5p impairs CAF-regulated angiogenesis and may sidestep this unwanted outcome.

Despite the promising effect of miR-425-5p in hampering BC progression, many difficulties must still be overcome before we can use miRNAs therapeutically. Limitations mainly concern degradation by nucleases and non-specific delivery to target tissues leading to off-target effects [62]. Although these challenges can be faced through different approaches, such as chemical modification reducing miRNA degradation, or the use of specific carriers, like aptamers for miRNA-specific delivery [63, 64], many more steps forward must be taken before we can translate miRNAs to the clinical scenario.

**Fig. 7:**
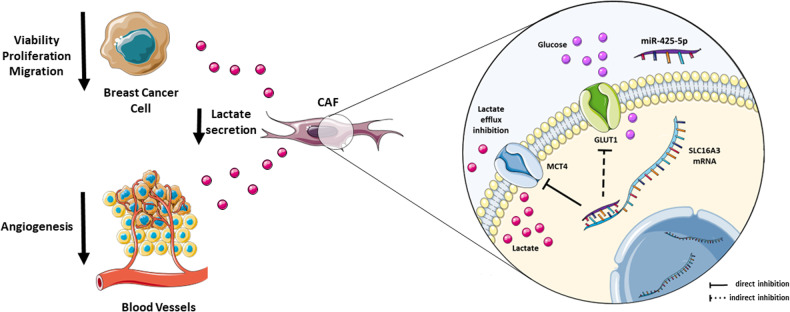
Schematic representation of miR-425-5p’s mechanisms of action. MiR-425-5p alters the metabolism of breast Cancer-associated Fibroblasts (CAFs) by downregulating MCT4 and indirectly reducing GLUT1, leading to decreased lactate extrusion. The interaction between miR-425-5p reprogrammed CAFs and breast cancer epithelial cells has a significant impact on breast cancer cell viability, proliferation, and migration. Angiogenesis is also influenced. This graphical abstract was drawn by using and adapting pictures from Servier Medical Art (Smart.Servier.com), provided by Servier, licensed under a Creative Commons Attribution 3.0 unported license (http://creativecommons.org/licenses/by/3.0/).

In conclusion, we demonstrated here that miR-425-5p reprograms the metabolism of breast cancer CAF’s through downregulation of MCT4 and reduction of lactate extrusion. The cross-talk between miR-425-5p reprogrammed CAFs and BC epithelial cells has a profound impact on the viability, proliferation, migration, and death of BC cells; angiogenesis is also affected (Fig. 7). Our findings thus support the importance of the metabolic symbiosis of BC cells and the surrounding stroma opening new scenarios for future BC treatment.

## Materials and methods

### Cell lines and reagents

Primary cancer-associated fibroblasts (CAFs) and normal fibroblasts (NFs) were derived from surgical resections provided by Clinica Mediterranea S.p.A. (Naples, Italy). Samples were collected according to the declaration of Helsinki and each subject signed an informed consent before participating in the study. The study was approved by the Research Ethics Committee of the University of Naples Federico II no. 119/15ES1. Continuous cell lines were from ATCC. CAFs and NFs were grown in DMEM/F12 (Sigma-Aldrich, Merck), MDA-MB-231 cells were grown in DMEM (Sigma-Aldrich, Merck), and BT-549 in RPMI-1640 (Sigma-Aldrich, Merck). Both media were supplemented with 10% heat-inactivated fetal bovine serum (Sigma-Aldrich, Merck), and 1% antibiotic-antimycotics (Life Technologies). MS5 cells were grown in RMPI supplemented with 20% heat-inactivated fetal bovine serum and 1% antibiotic-antimycotics. HUVECs were grown in EBM-2 endothelial cell growth Basal Medium supplemented with EGMTM-2 Endothelial SingleQuots Kit (Lonza Biosciences). Cell cultures were maintained at 37 °C in an atmosphere containing 5% CO_2_. Organoids were isolated from breast cancer surgical resections as previously described [65, 66]. Sodium L-lactate was supplied by Sigma Aldrich and used at a final concentration of 1 mM. All cells were routinely tested for mycoplasma contamination using a Mycoplasma PCR detection kit (Applied Biological Materials Inc. abm # G238).

### Protein extraction and Western blot analysis

Total protein extraction and Western blot analysis were performed, as previously discussed [67, 68]. For MCT4 analysis, cell membrane proteins were isolated using the Subcellular Protein Fractionation Kit for Cultured Cells (Thermo Fisher Scientific) according to the manufacturer’s protocol. Different ice-cold buffers were employed to sequentially separate cytoplasmatic, membrane, soluble nuclear, chromatin-bound nuclear, and cytoskeletal extracts. The following antibodies were used: anti-MCT4 (Santa Cruz Biotechnology and GeneTex sc-376140), anti-GLUT-1 (Santa Cruz Biotechnology sc-377228), anti-Na^+^/K^+^ ATPase (Santa Cruz Biotechnology, sc-48345), and anti-β-actin (Sigma-Aldrich- clone AC-15, ascites fluid). Secondary antibodies were goat anti-rabbit and anti-mouse IgG (ImmunoReagents, INC GtxRB-003-DHRPX, GtxMu-003-DHRPX). Protein levels were quantified by densitometric analysis via ImageJ software. Fold change was calculated over control.

### Cell transfection

miRNA mimic and miRNA inhibitor transient transfections were carried out using Oligofectamine (Invitrogen) with 100 nM pre-miR-425-5p and scrambled oligonucleotide control, or with 200 nM anti-miR-425-5p and anti-scrambled (Ambion). Plasmid transfection in MS-5 (2500 ng of SLC16A3 tGFP-tagged supplied by Origene CAT#: RG211987) was carried out using Lipofectamine 3000 reagent (Invitrogen).

### Immunofluorescence

Fibroblasts were seeded at a density of 5 × 10^4^ cells on sterile microscope slides. After one day of culture, cells were fixed and permeabilized with freshly prepared methanol/acetone (1:1) at −20 °C. After blocking with 1% BSA in PBS at room temperature, the primary antibody (MCT4, Santa Cruz Biotechnology) was incubated for 1 h at RT. Then, cells were incubated for 30 min at RT with Cyanine-3 fluorochrome-conjugated anti-mouse secondary antibodies (Jackson ImmunoResearch- 209-165-082). Finally, slides were stained with DAPI (10 μg/ml) (BD Biosciences) and mounted with PBS/glycerol mix (1:1).

### Cloning of SLC16A3 3′UTR target site for miR-425-5p and Luciferase assay

The putative binding site for the miR-425-5p seed sequence of SLC16A3 mRNA was predicted with a bioinformatics approach (TargetScan 7.2 http://www.targetscan.org/ and miRanda target prediction algorithms http://www.microrna.org/microrna), searching conserved 8-mer, 7-mer, and 6-mer sites that matched the seed region of the miRNA. 38 bp of SLC16A3 3′ UTR was cloned by using XbAI and SACI restriction enzymes in pmirGLO plasmid (supplied by Promega) and with the use of the following oligos: 5′CTAGAGCAGATCCACTCTGGAATGACACGGTTCCCACCCCGCCACGAGCT3′; and 5′ CGTGGCGGGGTGGGAACCGTGTCATTCCAGAGTGGATCTGCT 3′- [69, 70]. The seed sequence was in position 18-25 of the oligo without restriction sites.

MDA-MB-231 cells were seeded at a density of 2 × 10^3^ cells per well (96-well) and co-transfected with 100 ng of pmirGLO vector containing SLC16A*3* 3′UTR and 100 nM of miR-425-5p or scrambled control (Ambion), using Lipofectamine 3000 (Invitrogen). Upon 72 h of transfection, cells were lysed with passive lysis buffer (Promega) and reporter gene activity was quantified with Dual-Luciferase Reporter Assay (Promega), according to the manufacturer’s protocol.

### Conditioned media collection

CAFs were seeded (5 × 10^5^) and transfected with miR-425-5p for 72 h. Afterwards, they were washed twice with ice-cold 1x PBS (Sigma) and incubated with fresh DMEM F12 (Sigma) for 7 h. Conditioned media were collected and centrifuged to eliminate dead cells and debris.

### Caspase assay and viability assay

MDA-MB-231 cells were seeded in triplicates at a density of 2 × 10^3^ per well (96-well) and simultaneously treated with conditioned media (1:1 ratio with fresh DMEM) and eventually L-lactate [1 mM] for 48 h. Caspase-3 activity was analyzed by Caspase-Glo 3/7 Assay (Promega). Cell viability was analyzed by CellTiter 96 AQueos One Solution Cell Proliferation Assay (MTS), supplied by Promega as previously reported [69, 71]. For organoids, clear bottom 96-well plates were coated with 30 µl of BME before the addition of 120 µl of organoids suspension. After 7 days, CM of CAFs transfected with miR425-5p, alone or in combination with 1 mM of lactate, diluted in a 1:1 ratio with organoid media were added in triplicate. To evaluate the effects of CM treatment on organoid viability, 20 µl of Celltiter 96 was added to each point after 48 h of treatment.

### Migration assay

Migration assays were carried out with 6.5 mm Transwell permeable support with an 8-μm pore polycarbonate membrane (Corning), as previously reported [71]. Cells were resuspended at a density of 1 × 10^5^ in a serum-free medium and seeded in triplicates in the upper chamber. The lower chamber of the transwell was filled with conditioned medium, supplemented by 10% FBS. After 24 h, transwells were stained with 0.1% crystal violet in 25% methanol. Non-migrated cells were scraped off the top of the transwell with a cotton swab. The percentage of migrated cells was evaluated by eluting crystal violet with 1% sodium dodecyl sulfate (SDS) and reading dye absorbance at λ = 570 nm.

### Tube formation assay

HUVECs were seeded on Matrigel-coated plates at a density of 3.5×10^4^ in 48-well plates. They were transfected with 100 nM) miR-425-5p or scrambled control. Otherwise, they were treated with fibroblast-derived CM and subsequently incubated overnight. The plates were photographed, and the pictures were analyzed using ImageJ with the Angiogenesis Analyzer tool.

### Lactate concentration measurement

Intracellular and extracellular L-lactate concentration was detected using the L-lactate Assay colorimetric kit (Abcam), according to the manufacturer’s protocol.

### RNA extraction and Real-Time PCR

Total RNA was extracted with TRIzol reagent (Invitrogen), according to the manufacturer’s protocol. Reverse transcription of total miRNAs was performed using miScript II Reverse Transcription Kit (Qiagen), whereas for mRNA retrotranscription we used SuperScript III Reverse Transcriptase (Thermo Fisher Scientific). Quantitative real-time PCR was performed with specific primers for MCT4 and β-actin (as internal reference gene) supplied by IDT and with iQTM SYBR Green Supermix (Bio-Rad). Regarding miR-425-5p and U6 snoRNA (as internal reference gene), we used specific primers supplied by Qiagen and miScript SYBR Green PCR Kit (Qiagen).

### CAF metabolic analysis

Cellular glycolysis was measured using a Seahorse XF Glycolytic Rate Assay on the Seahorse XFe96 extracellular flux analyzer (Seahorse Bioscience, Agilent Technologies, Santa Clara, CA, USA). For each tested cellular condition, cells were plated in eight wells of a Seahorse 96‐well plate, 24 h before the assay, with a seeding density of 1 × 10^4^ cells/well. Fresh XF medium (non-buffered DMEM medium, containing 10 mM glucose, 2 mM L‐glutamine, and 1 mM sodium pyruvate) was prepared on the day of the assay and used to resuspend the plated cells after removing their medium. The plate was then incubated at 37 °C in a non-CO_2_ incubator for 1 h before the start of the assay. ECAR and OCR were measured under basal conditions and after the injection of 0.5 μM rotenone (Rot - Complex 1 inhibitor, allows to inhibit mitochondrial activity and, thus, mitochondrial oxygen consumption), and 50‐mM 2‐deoxy‐D‐glucose (2‐DG – a glucose analog able to act as glycolysis inhibitor). Each change of OCR and ECAR, both at basal conditions and after each compound’s injection, was measured 3 times and the average values were used to calculate glycolytic feature. Data Analysis was carried out using the Seahorse Analytics software (Agilent, Santa Clara, CA, United States). A T-test was applied to evaluate results’ significance.

### CAF co-culture experiments

CAFs (8 × 10^4^ cells) were transfected with miR-425-5p and scrambled sequences and after 48 h 12 × 10^4^ CellTracker™ Green CMFDA Dye-labelled BT-549 cells were added. BT-549 cell viability was measured by FACS analysis after 72 h. For cell cycle analysis the same protocol was performed without the addition of the doxorubicin.

### Statistical analysis

All data were analyzed for significance using GraphPad Prism 8.0.2 (San Diego, CA, USA). Continuous variables are given as mean ± SD or ± SEM. Statistical significance (*p* < 0.05, *p* < 0.01, *p* < 0.001) was assessed using Student’s t-test (for comparisons between two groups), and one-way or two-way ANOVA coupled with Tukey’s post-hoc testing (for multiple comparisons).

### Supplementary information

Supplemental Material

Original Data File

## Data Availability

The datasets generated during and/or analysed during the current study are available from the corresponding author upon reasonable request.
