# Supplementary material for: MCT4-driven CAF-mediated metabolic reprogramming in breast cancer microenvironment is a vulnerability targetable by miR-425-5p
Source: Cell Death Discov. 2024 Mar 14;10:140. doi: 10.1038/s41420-024-01910-x (PMC10940713; doi:10.1038/s41420-024-01910-x)
Supplement: Supplementary file 1 — Supplemental Material [file 41420_2024_1910_MOESM1_ESM.docx]

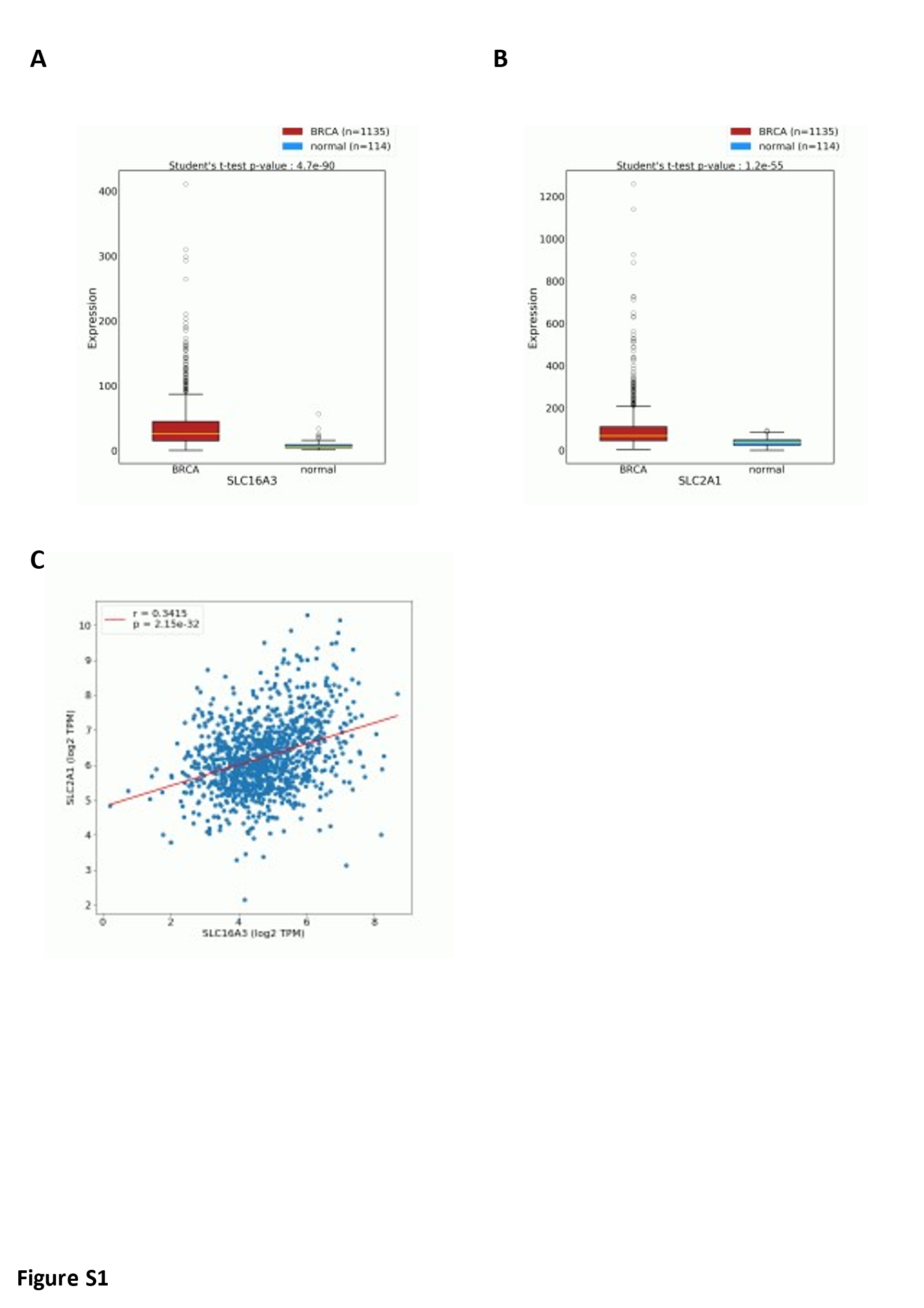


**Figure S1. MCT4/GLUT1 expression analysis in BC**

Expression profile of SLC16A3 (MCT4) (A) and SLC2A1 (GLUT1) (B) in Breast Invasive Carcinoma (BRCA) is shown. Plot pair-wise gene expression correlation analysis between SLC16A3 and SLC2A1 in BRCA (C). Source https://oncodb.org.


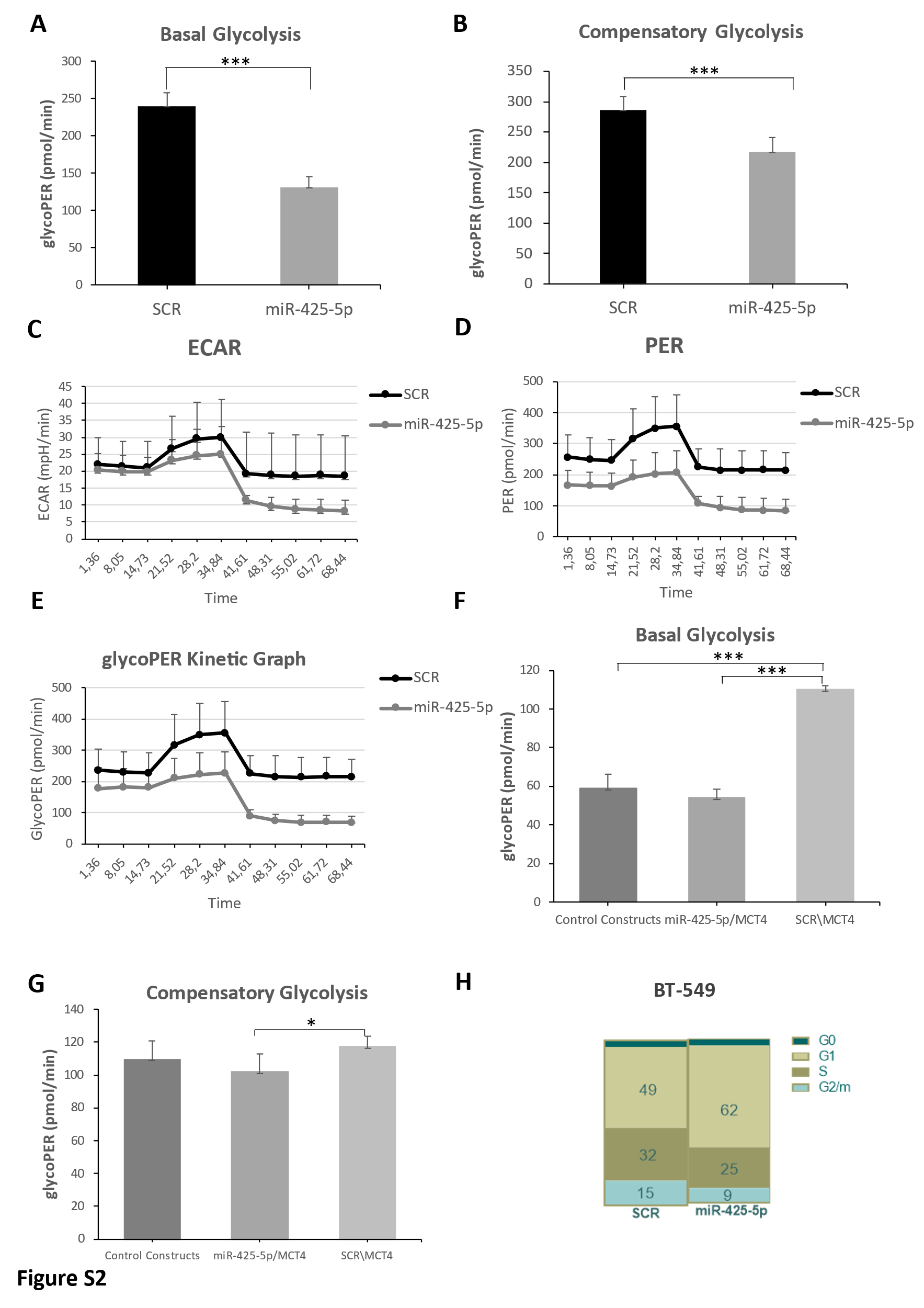


**Figure S2. Fibroblasts’ metabolic changes mediated by miR-425-5p.**

GlycoPER quantification in CAFs transfected with miR-425-5p (**A,B**) and in MCT4-transfected MS-5 cells with or without miR-425-5p overexpression (**F,G**) during basal Glycolisis (**A,F**) and compensatory Glycolisis (**B,G**). Data are presented as a mean of biological duplicates of eight technical replicates ± SD over control. Metabolic changes in MS-5 cells with or without miR-425-5p overexpression, as assessed by glycolytic proton efflux rate (glycoPER) kinetics (**C**), proton efflux rate (PER) **(D)**, and extracellular acidification rate (ECAR) (**E**), are assessed. Data are presented as a mean of eight technical replicates ± SD over control. Cell cycle analysis of CMDFA-labelled BT-549 is shown upon co-culture with CAFs overexpressing miR-425-5p/scrambled sequence **(H)**. Data are presented as a mean of biological duplicates of two technical replicates ± SD over control. * p < 0.05; ***p < 0.001.


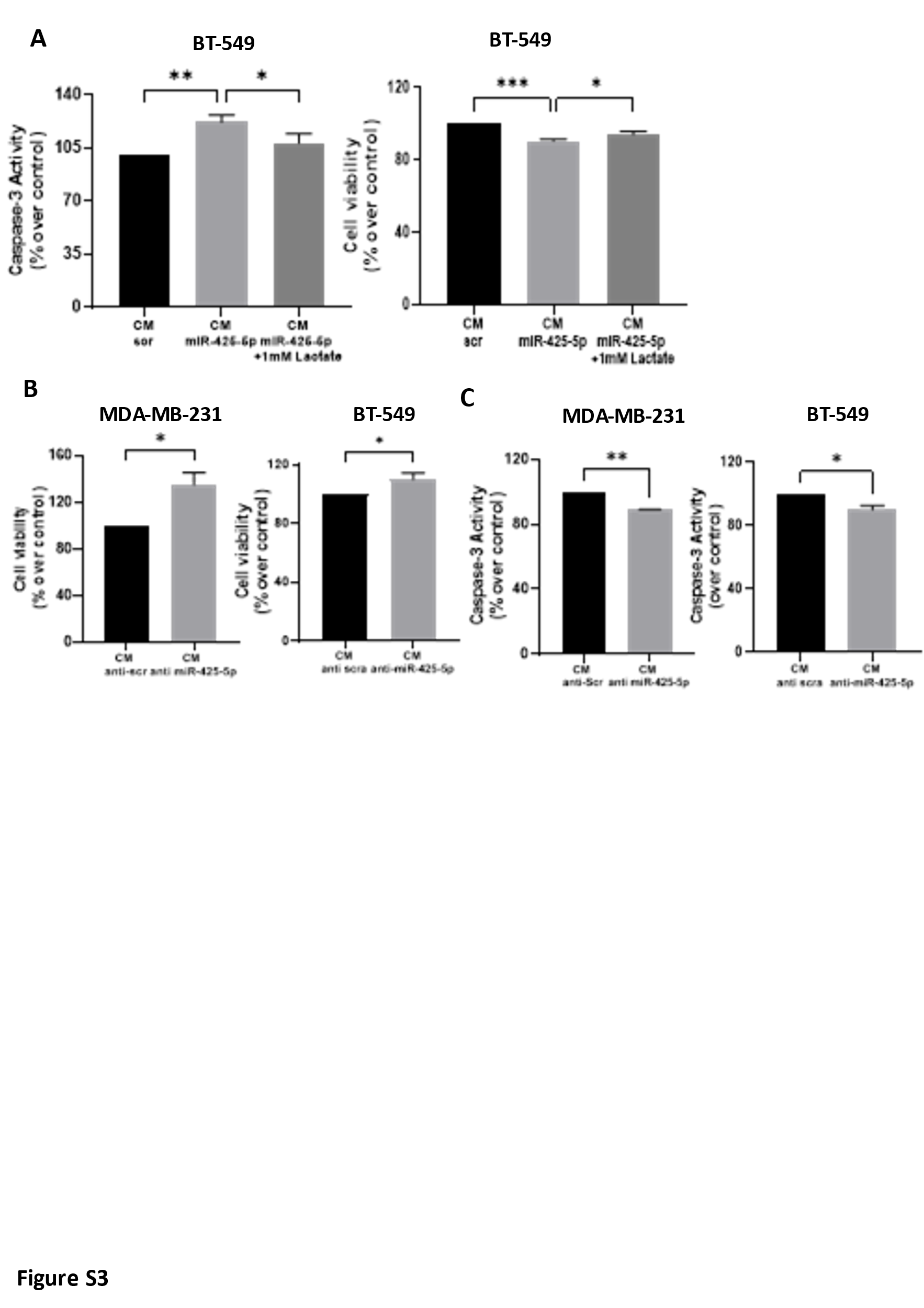


**Figure S3. Cell viability and caspase 3-activation mediated by conditioned medium from miR-425-5p-overexpressing CAFs or miR-425-5p downregulated NFs on BC cells.**

Cell viability and caspase 3-activation **(A)** of BT-549 cells treated with conditioned medium from CAFs overexpressing miR-425-5p or a scrambled sequence. 1mM of lactate was used in rescue experiments. Cell viability and caspase 3-activation **(B)** of BT-549 and MDA-MB-231 cells treated with conditioned medium from NFs overexpressing anti-miR-425-5p.
