# Supplementary material for: MCT4-driven CAF-mediated metabolic reprogramming in breast cancer microenvironment is a vulnerability targetable by miR-425-5p
Source: Cell Death Discov. 2024 Mar 14;10:140. doi: 10.1038/s41420-024-01910-x (PMC10940713; doi:10.1038/s41420-024-01910-x)
Supplement: Supplementary file 2 — Original Data File [file 41420_2024_1910_MOESM2_ESM.pdf]

FIG 1 A MEMBRANE PROT. FRACTION

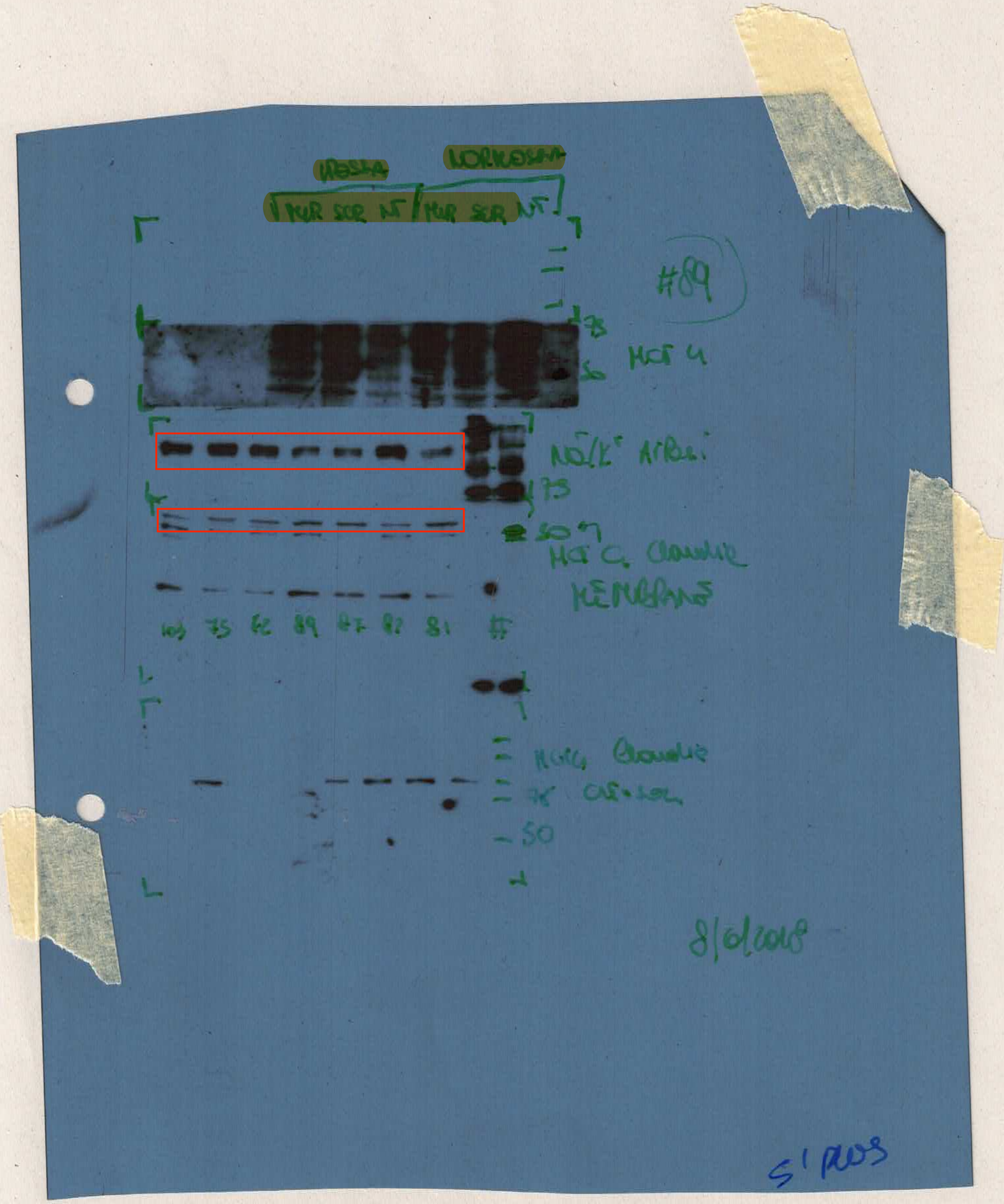

FIG. 2 B PANNELLO I e II (72h) CAF5

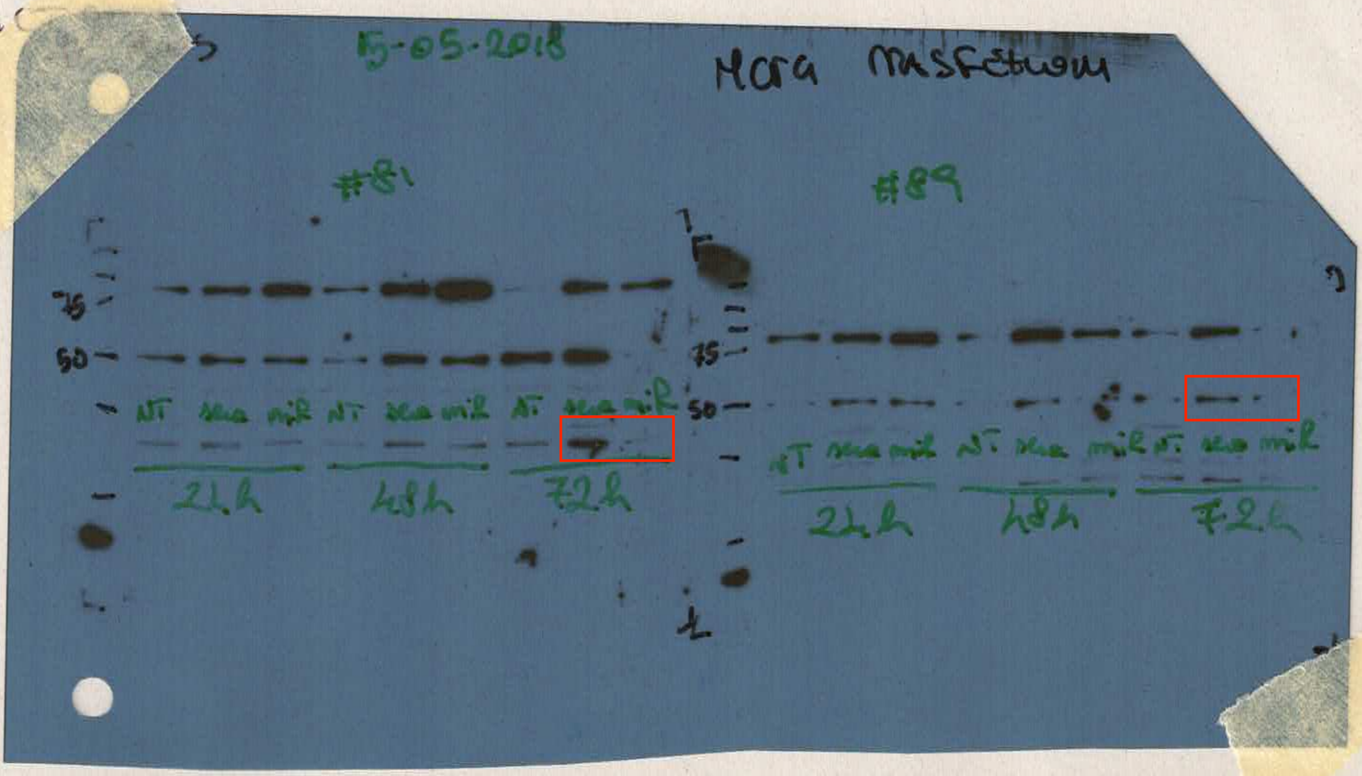

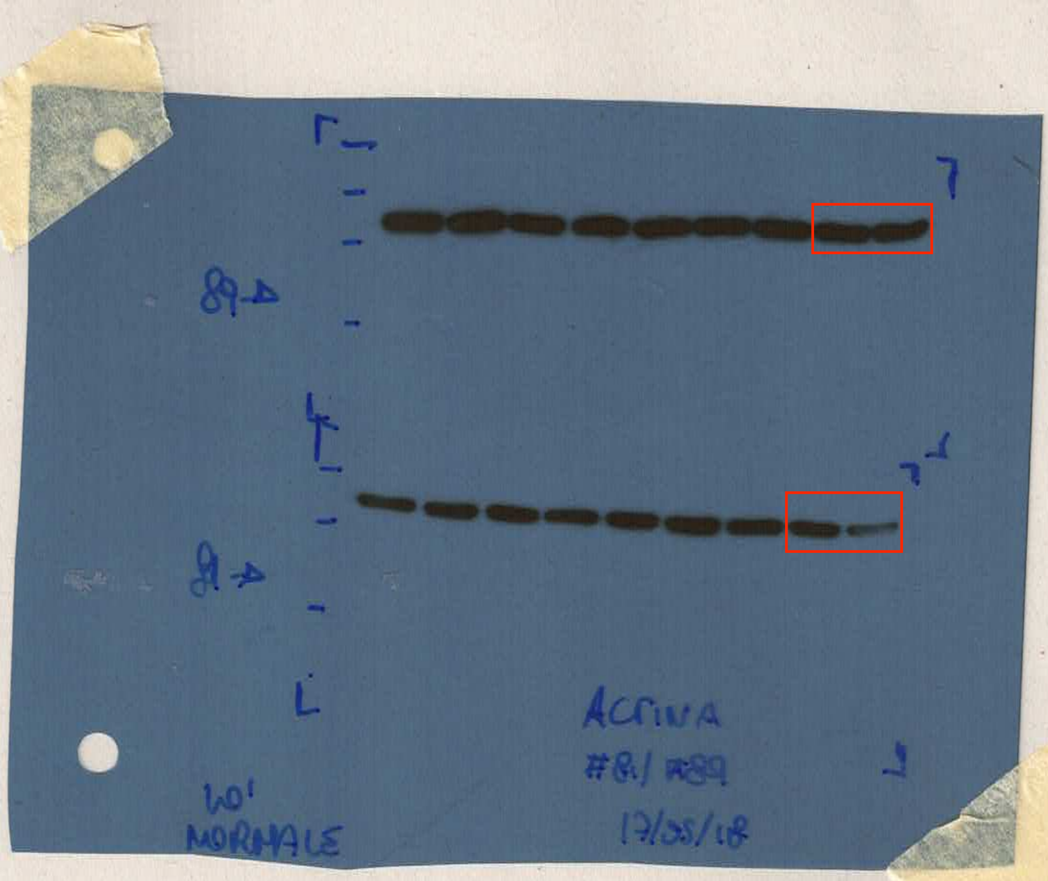

FIG. [illegible] B PANNELLO III 72h CAFs

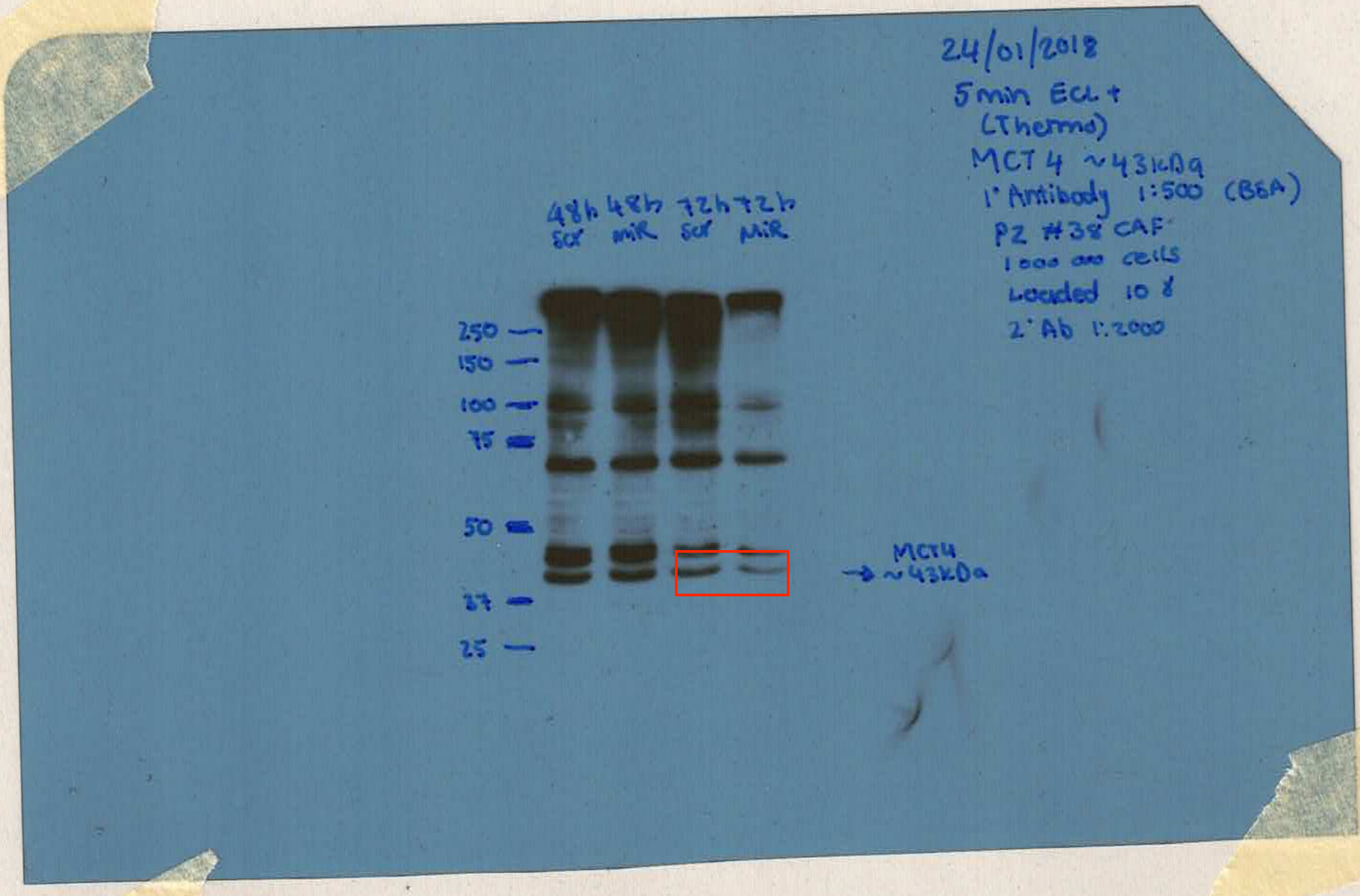

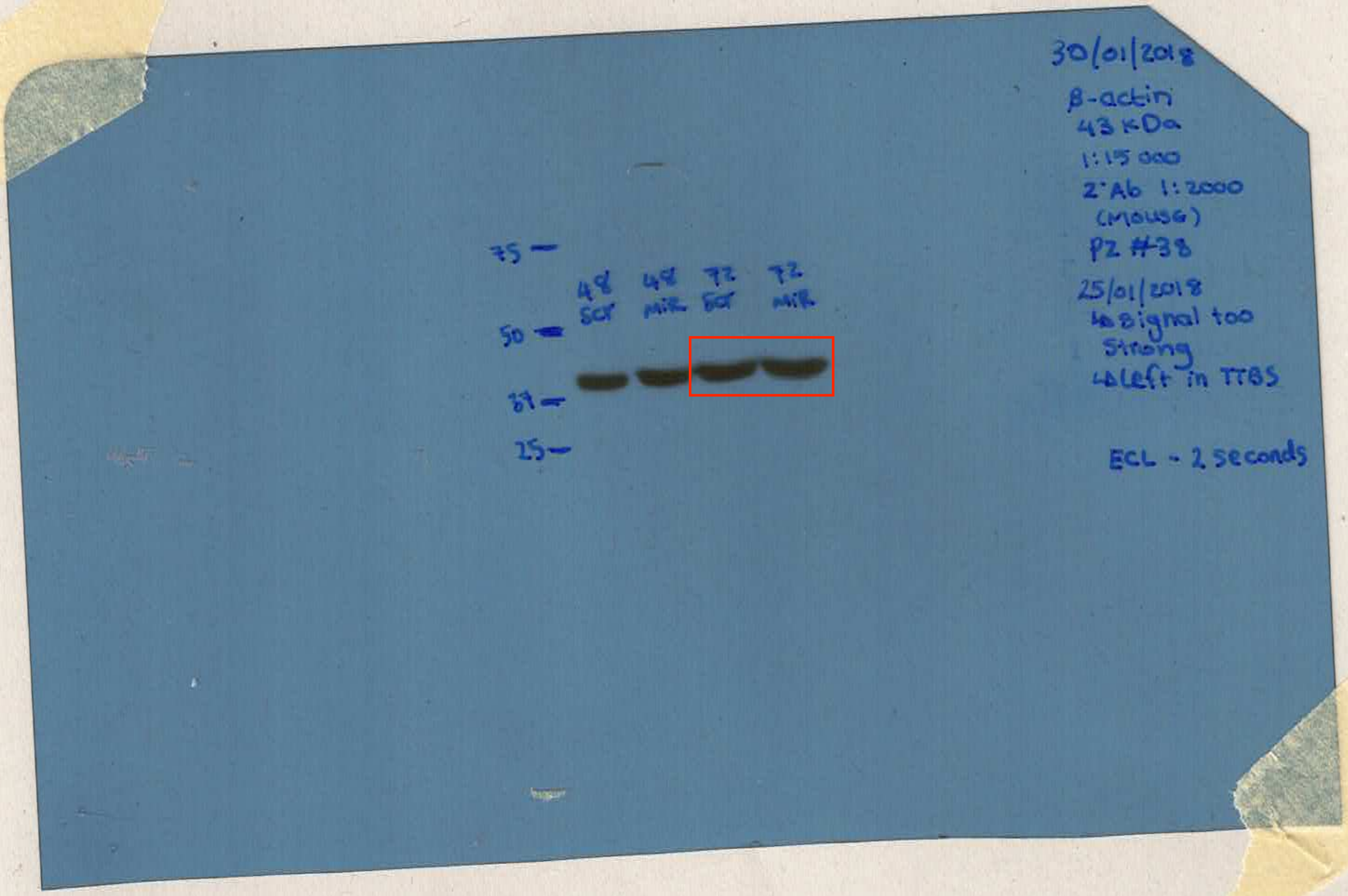

FIG. 2 B PANNELLO III CAFs

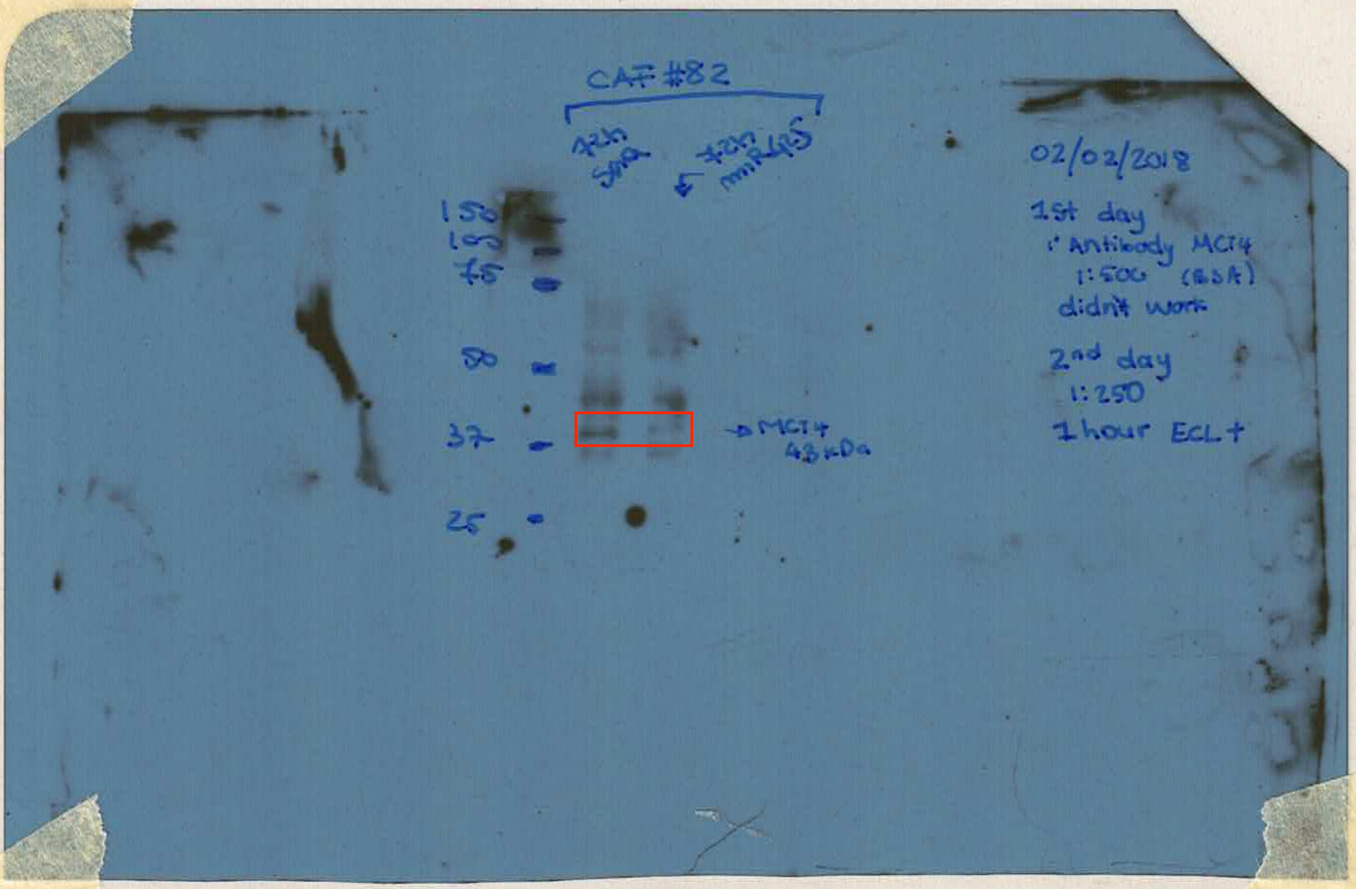

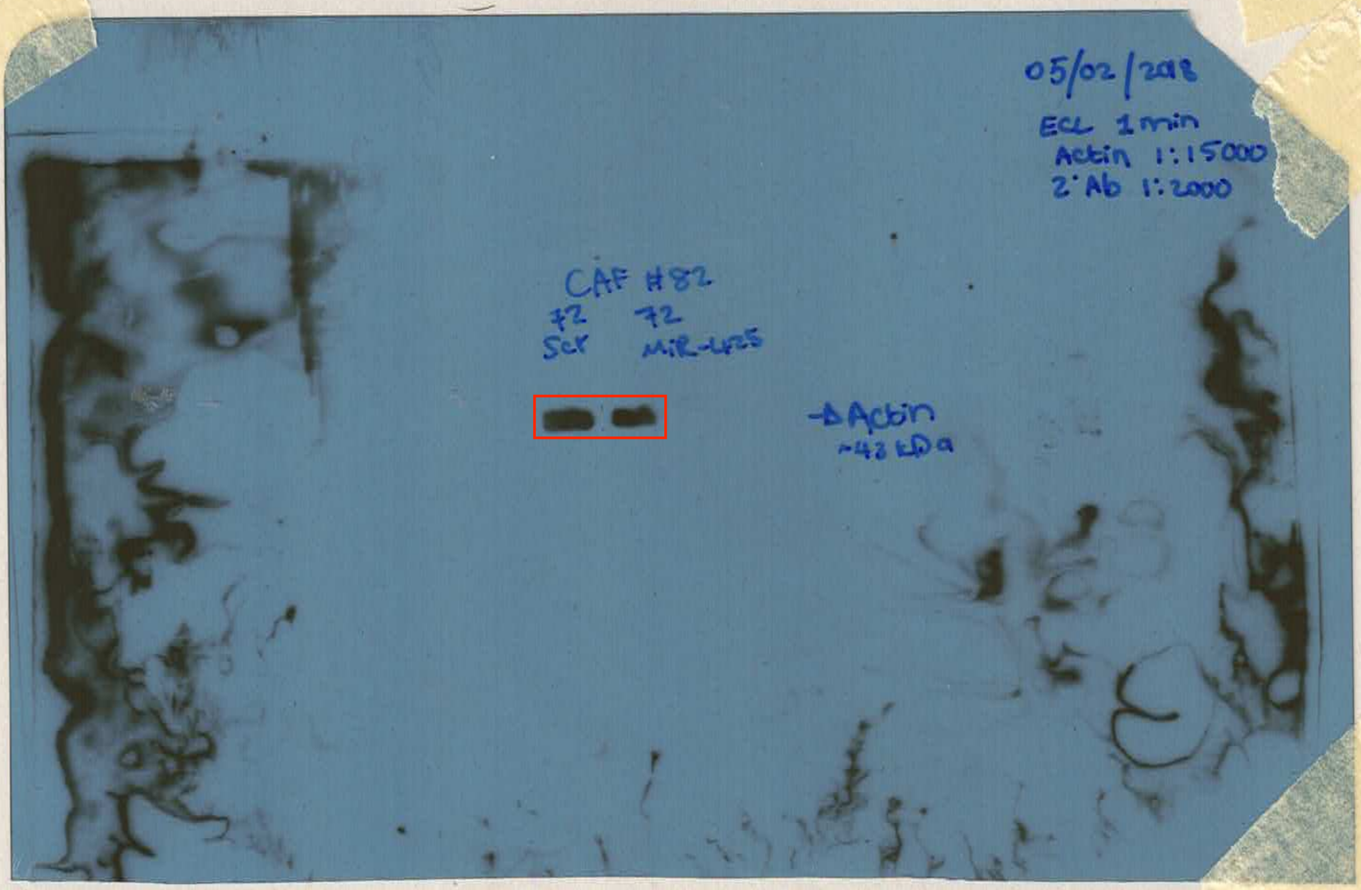

FIG. 2B PANNELLO NFs V ANTIMIR 48h

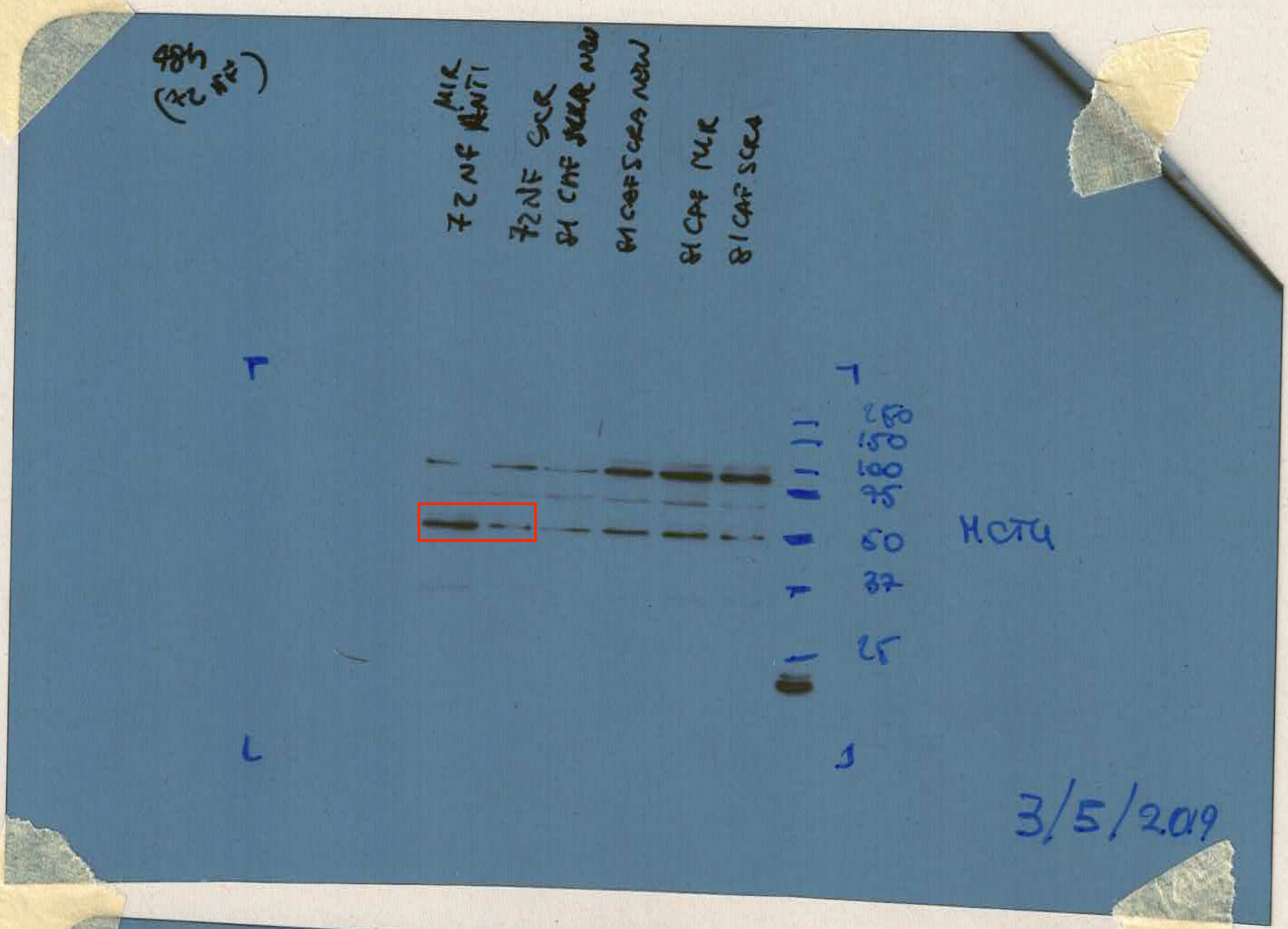

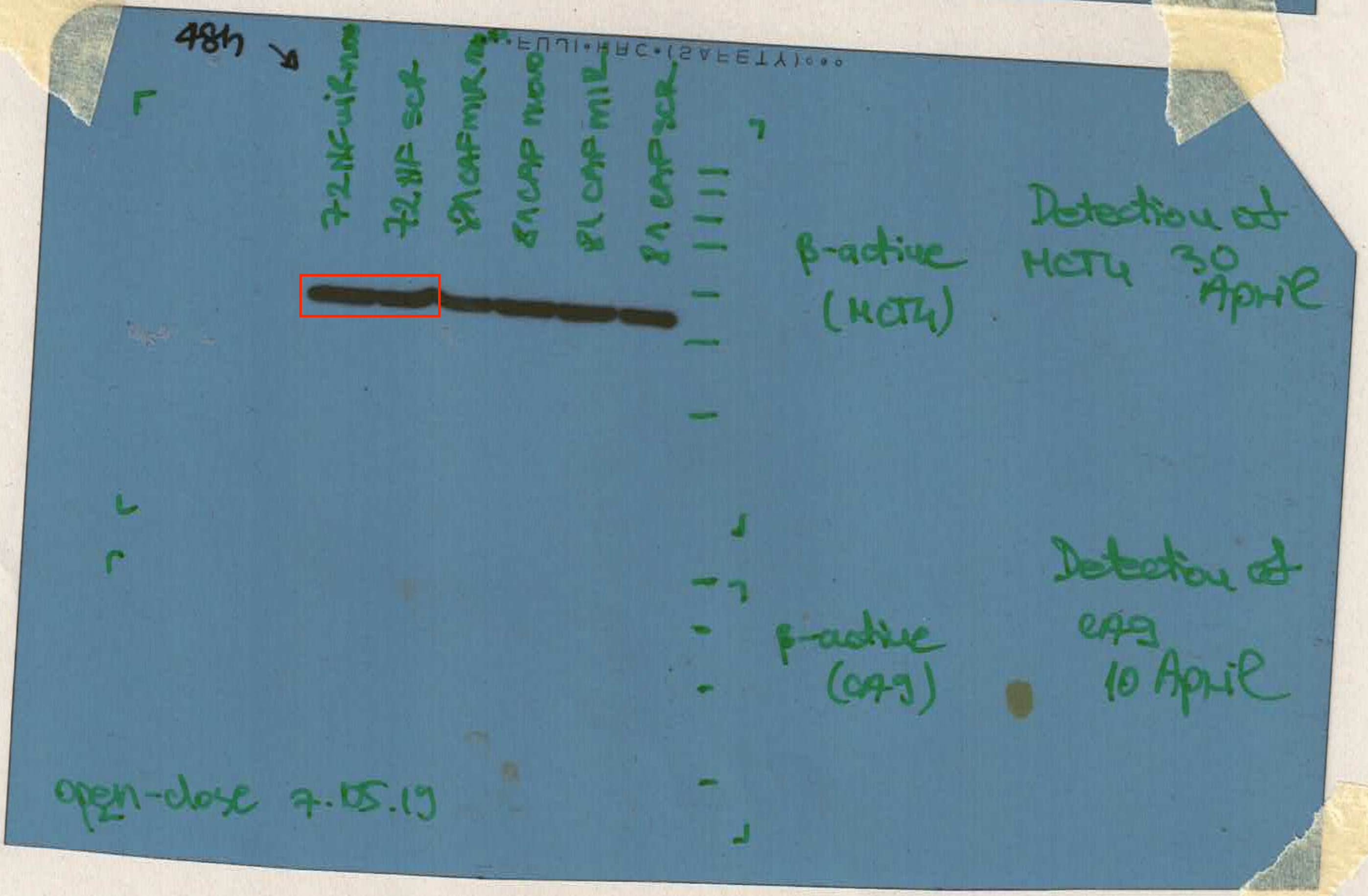

2B PANNELLO NFs VI

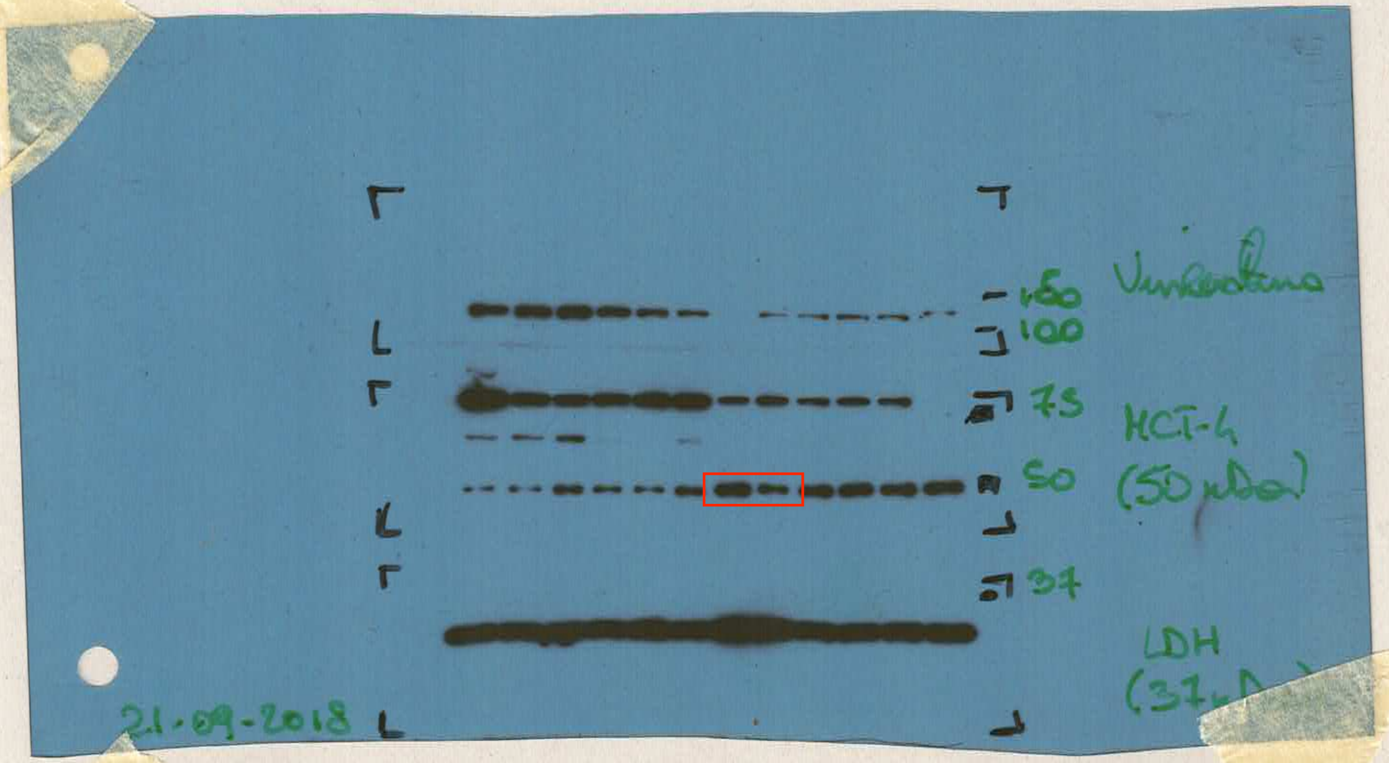

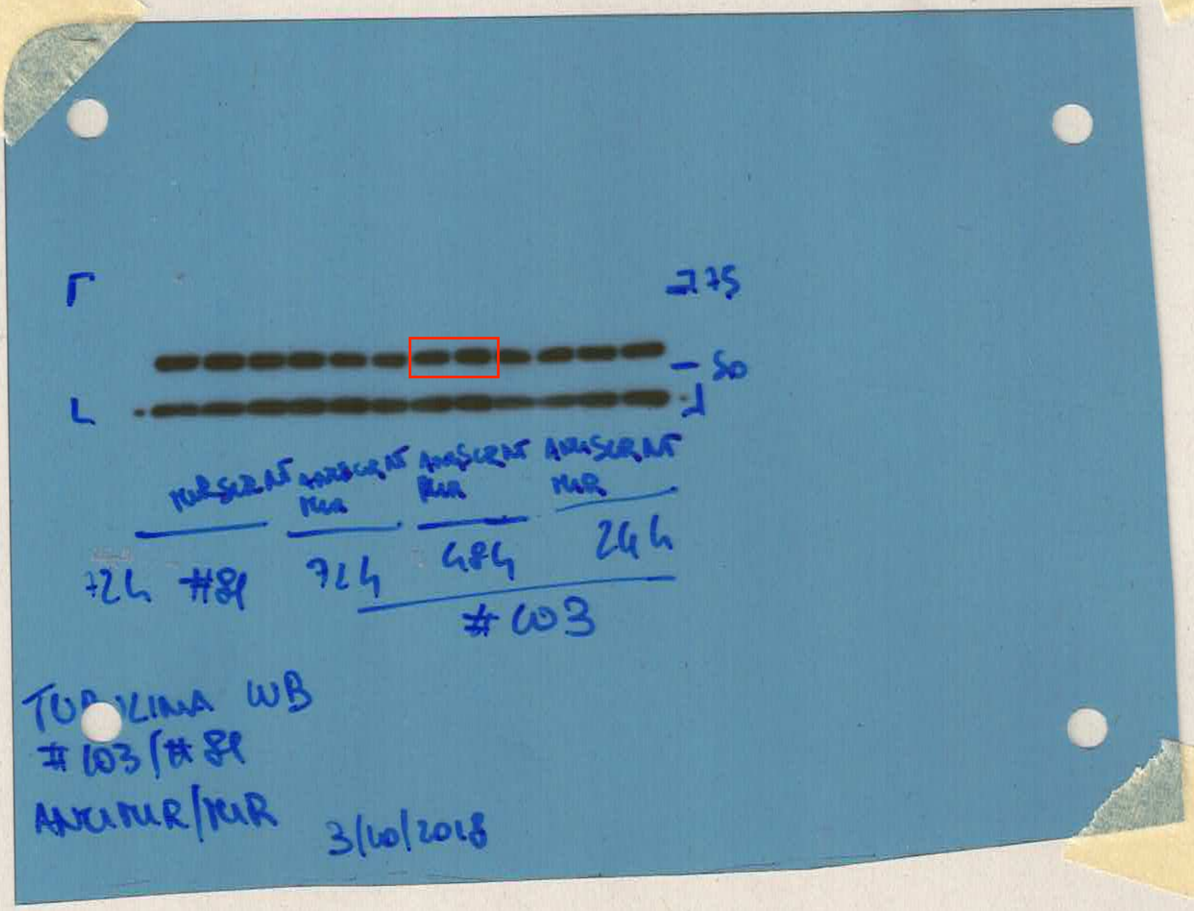

FIG. 3 C

Ibridato un vecchio filtro su cui era già stata ibridata l'actina precedentemente (ancora visibile)

Se non ero stesso [illegible] secondario

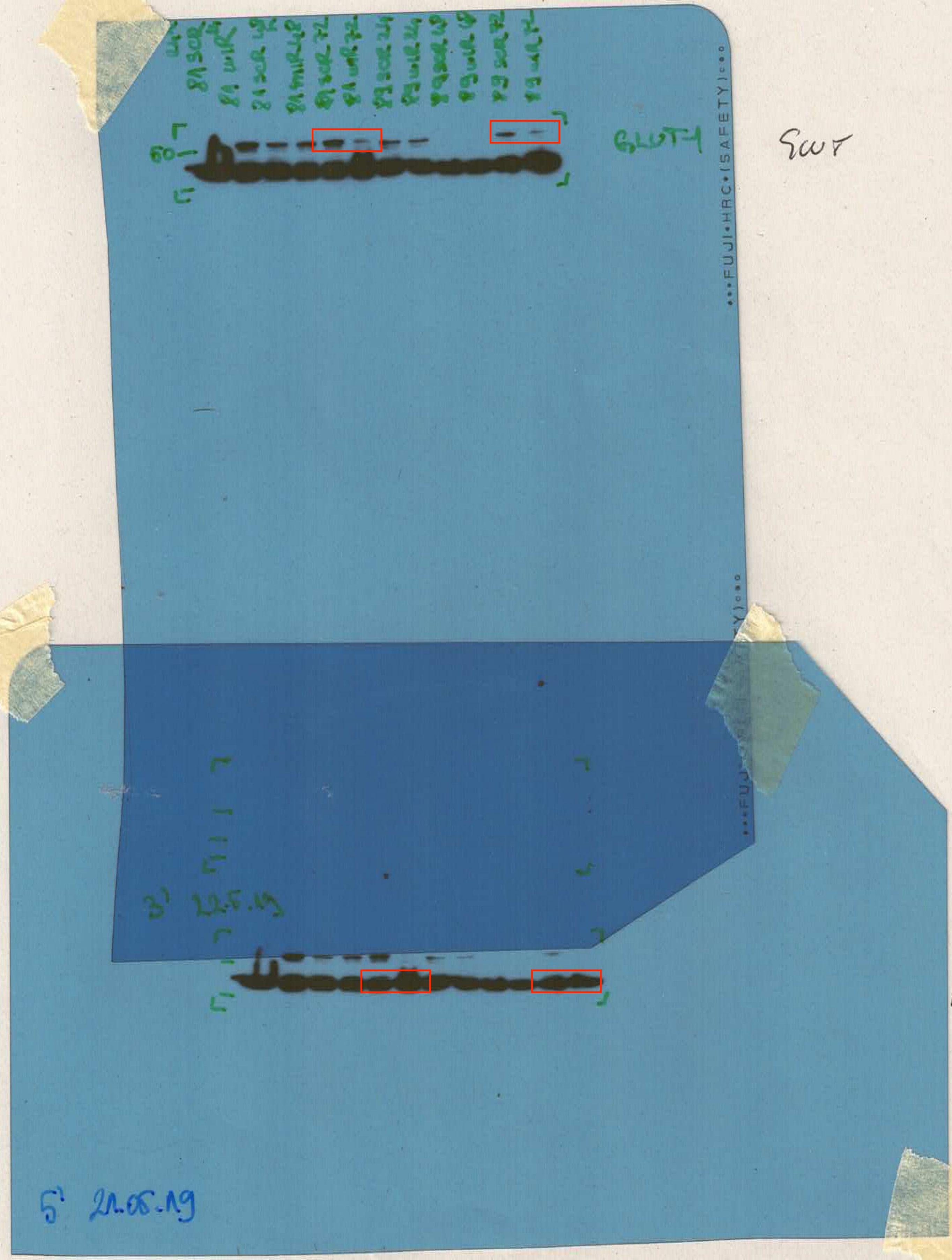

Glut

Actin
